# A systematic review and meta-analysis of the aetiological agents of non-malarial febrile illnesses in Africa

**DOI:** 10.1371/journal.pntd.0010144

**Published:** 2022-01-24

**Authors:** Martin Wainaina, David Attuy Vey da Silva, Ian Dohoo, Anne Mayer-Scholl, Kristina Roesel, Dirk Hofreuter, Uwe Roesler, Johanna Lindahl, Bernard Bett, Sascha Al Dahouk

**Affiliations:** 1 Department of Biological Safety, German Federal Institute for Risk Assessment, Berlin, Germany; 2 Department of Veterinary Medicine, Freie Universität Berlin, Berlin, Germany; 3 International Livestock Research Institute, Nairobi, Kenya; 4 University of Prince Edward Island, Charlottetown, Canada; 5 Institute for Animal Hygiene and Environmental Health, Freie Universität Berlin, Berlin, Germany; 6 Department of Clinical Sciences, Swedish University of Agricultural Sciences, Uppsala, Sweden; 7 Department of Medical Biochemistry and Microbiology, Uppsala University, Uppsala, Sweden; 8 Department of Internal Medicine, RWTH Aachen University Hospital, Aachen, Germany; ICIPE: International Centre for Insect Physiology and Ecology, KENYA

## Abstract

**Background:**

The awareness of non-malarial febrile illnesses (NMFIs) has been on the rise over the last decades. Therefore, we undertook a systematic literature review and meta-analysis of causative agents of non-malarial fevers on the African continent.

**Methodology:**

We searched for literature in African Journals Online, EMBASE, PubMed, Scopus, and Web of Science databases to identify aetiologic agents that had been reported and to determine summary estimates of the proportional morbidity rates (PMr) associated with these pathogens among fever patients.

**Findings:**

A total of 133 studies comprising 391,835 patients from 25 of the 54 African countries were eligible. A wide array of aetiologic agents were described with considerable regional differences among the leading agents. Overall, bacterial pathogens tested from blood samples accounted for the largest proportion. The summary estimates from the meta-analysis were low for most of the agents. This may have resulted from a true low prevalence of the agents, the failure to test for many agents or the low sensitivity of the diagnostic methods applied. Our meta-regression analysis of study and population variables showed that diagnostic methods determined the PMr estimates of typhoidal *Salmonella* and Dengue virus. An increase in the PMr of *Klebsiella* spp. infections was observed over time. Furthermore, the status of patients as either inpatient or outpatient predicted the PMr of *Haemophilus* spp. infections.

**Conclusion:**

The small number of epidemiological studies and the variety of NMFI agents on the African continent emphasizes the need for harmonized studies with larger sample sizes. In particular, diagnostic procedures for NMFIs should be standardized to facilitate comparability of study results and to improve future meta-analyses. Reliable NMFI burden estimates will inform regional public health strategies.

## Author summary

Previous systematic reviews have highlighted the research priorities of causative agents for non-malarial febrile illnesses by counting the number of publications attributed to an agent. However, proportional morbidity rates are calculated by dividing the number of cases with a specific disease (numerator) by the total number of diagnosed fever cases (denominator) and are better indicators of the relative importance of aetiological agents in a population. Therefore, we present the leading causes of non-malarial febrile illnesses in African patients in both healthcare and community settings. Preference is given to HIV-negative patients when data could be found. We also determined summary estimates of *Brucella* spp., Chikungunya virus, Dengue virus, *Haemophilus* spp., *Klebsiella* spp., *Leptospira* spp., non-typhoidal *Salmonella* spp., typhoidal *Salmonella* spp., *Staphylococcus* spp., and *Streptococcus* spp.

The wide array of aetiological agents causing febrile illnesses on the African continent does not only complicate malaria control programs but may also hamper response to epidemic and pandemic illnesses such as Ebola and COVID-19. The harmonisation of diagnostics and study designs will reduce between-study differences, which may result in better estimates of disease burden on the continent and in the different African regions. This information is important for Pan-African surveillance and control efforts.

## Introduction

There has been tremendous progress in the control of fevers that are attributable to *Plasmodium falciparum* in Africa [1]. So far, five countries on the continent have been certified as malaria-free [2]. This success is largely due to intensive control strategies such as the use of insecticide-treated bed nets, indoor residual spraying, and the use of artemisinin-based combination therapy for the treatment of clinical malaria cases [3]. Fever however remains commonly reported among patients in Africa and is even used as an indicator of health-seeking behaviour in many national health surveys [4]. Despite the widespread occurrence of non-malarial febrile illnesses (NMFIs) in various parts of the continent, the similarity of clinical features makes differential diagnosis difficult since pathognomonic signs are often missing [5]. This, along with inadequate laboratory diagnostic capacity [6], impedes good estimates of the burden of these infections. However, determining the burden of NMFIs promotes the establishment of targeted surveillance strategies, which in turn may help to reduce disease burden. As the malaria incidence reported in some African countries has recently increased [7], defining the burden of NMFIs will lessen complications in malaria control programs as well as raise awareness of their existence in communities [8,9]. Some NMFIs have also complicated the diagnosis of pandemic illnesses such as COVID-19 (SARS-CoV-2) and swine flu (H1N1), consequently posing serious public health threats in endemic areas [10,11].

Despite NMFI studies from Africa being extensively reviewed [12–17] and their research focus explored [18], there is a lack of information on estimates of disease burden as well as on important determinants of the occurrence of NMFIs in fever patients. Proportional morbidity rates (PMr), which are calculated by dividing the number of diagnoses attributable to an aetiologic agent by the total number of diagnoses in a study, may close this information gap [19].

We, therefore, conducted a systematic review and meta-analysis with the aim of identifying pathogens commonly occurring in fever patients in Africa. Furthermore, we estimated PMr associated with these agents, which is useful in setting public health priorities by illustrating the relative importance of diseases in a population. Finally, we determined population and study characteristics that influenced the PMr of the aetiologic agents by meta-regression analyses.

## Methods

**Fig 1:**
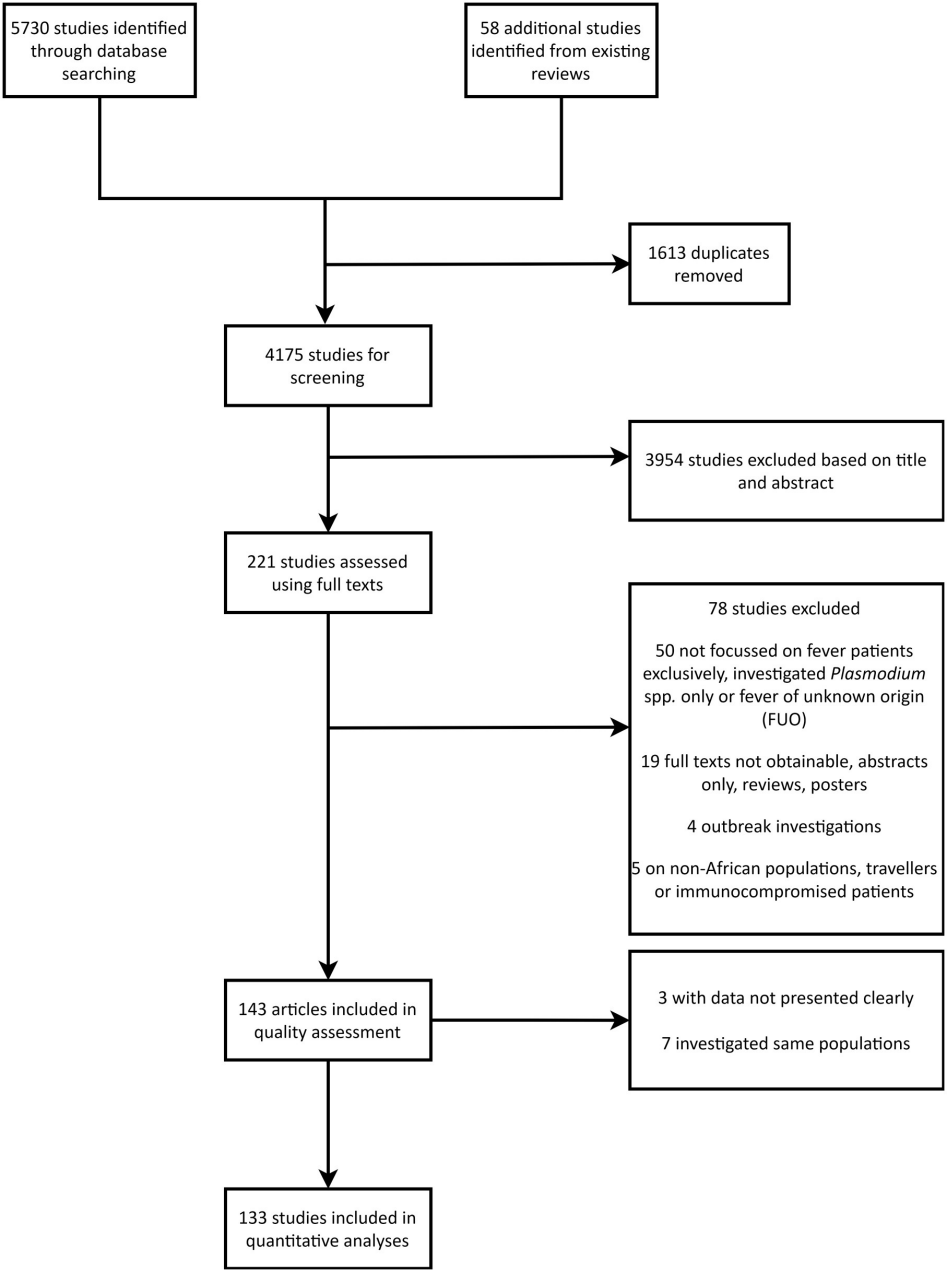
Systematic review process to select the studies relevant for meta-analysis of NMFIs in Africa.

The systematic literature review and meta-analysis was undertaken according to PRISMA guidelines [20]. The PRISMA flowchart in Fig 1 shows a summary of the selection process. Literature was retrieved from five databases, namely African Journals Online (AJOL), Embase, PubMed, Scopus, and Web of Science. To obtain unbiased results from the literature search, we used the following search terms: "undifferentiated" OR "unknown" OR "non*malaria*" AND "fever" OR "pyrexia" OR "hyperthermia" OR "febrile" AND "Africa". The syntax of search terms and Boolean operators are summarised in S1 Table. We set no time limit and allowed all languages. The searches were conducted between 22^nd^ November and 24^th^ December 2018. When full texts could not be found, MW contacted the study authors directly for a copy of the manuscript.

### Literature screening and reviewing

References and their abstracts were imported into EndNote software (Thomson Reuters, Philadelphia, PA, USA) and the duplicate records were removed. Article titles and abstracts were reviewed for eligibility using predefined inclusion and exclusion criteria by two of the co-authors independently (MW, DAVdS). In case of disagreement on whether a reference should be included or not, other co-authors (AMS, SAD) made the final decision. Observational studies in Africa focusing on humans with febrile illnesses that were initially undifferentiated, but aetiological agents were later determined by laboratory investigations were included. The following references were excluded: case reports, case series, books and book sections, grey literature, studies describing study populations not based in Africa, studies on travellers, studies on animals or insects, studies not focused on fever patients exclusively, studies investigating “fever of unknown origin” [21], studies not investigating fever agents, editorials and publications lacking original data, conference abstracts, conference proceedings, review articles, and outbreak investigations. We however included outbreak investigations that gave epidemiological descriptions, presenting information by demographic (e.g. gender, occupation, age), location, and/or temporal characteristics of the outbreaks [22].

Relevant articles not captured by our initial search but found in major reviews on the topic were added to the initial list of identified articles.

### Risk of bias assessment

We assessed the quality of the studies using the criteria outlined in the Critical Appraisal Skills Program (CASP) tool [23] and Alonso *et al*. [24]. The studies were scored using a simple scale to assess the risk of bias. The quality criteria were aimed at judging whether studies had a clearly focused topic, chose their study populations reasonably, defined and detected cases appropriately, analysed data robustly, and presented results and discussed them clearly (S1 Text). Results of the scoring are presented in the supporting information (S1 Fig).

### Data extraction, inclusion and extraction criteria

The authors jointly agreed on data to be extracted from the selected articles. Two reviewers added information from the full texts to an MS Excel file comprising the details described in Table 1.

**Table 1:** Variables extracted from the included studies and their detailed description.

Variable	Categories found in the included studies/description of the variables
Country of study *^†^	country(/ies) of study population
Study end date (year) ^†^	at the end of sample collection; the variable was used either as a linear or categorical variable of ten-year durations
Aetiologic (agents) number ^†^	whether one genus was investigated (single aetiology study) or more than one genus was investigated (multiple aetiologies study)
Study season	wet or dry season
Study design	cross-sectional, cross-sectional (paired sampling), historical study, cross-sectional (retrospective), surveillance, outbreak investigations, and longitudinal studies
Study setting ^†^	urban, rural and other (which comprised peri-urban, suburban, and semi-urban settings)
Place of recruitment for study participants ^†^	community and healthcare facility (included patients recruited from primary healthcare facilities, hospitals, research facilities, and temporary treatment units)
Population status ^†^	inpatients, outpatients, or both
Age of study participants	the range, interquartile range, or mean of the patients admitted to the studies
Minimum fever temperature for study admission	depending on the case definition of included studies
Location of fever measurement	oral, rectal, axillary, tympanic, or not recorded
Duration of fever	depending on the case definition of included studies
Sample size under study	number of samples investigated; when possible, only HIV negative populations were considered
Aetiologic agent tested	for each agent, the number of patients tested and the number/percentage of positive test results were documented
Sample(s) tested	serum, whole blood, cerebrospinal fluid, nasopharyngeal swabs and/or aspirates, oropharyngeal swabs, plasma, sputum, stool, pus, or urine
Diagnostic test(s) applied ^†^	direct detection (of organisms or antigens) through culture, microscopy, agglutination test, lateral flow assay, ELISA, polymerase chain reaction (PCR), or metagenomics; indirect detection (IgG and/or IgM antibodies, specific antibody class not mentioned, neutralising antibodies or metabolites) through agglutination test, complement fixation test, ELISA, lateral flow assay, neutralising antibody test, immunofluorescence assay, western blot, and metabolomics; a combination of direct and indirect detection methods was assumed when both were used to determine the final number of cases
Clinical signs associated with aetiologic agents	clinical signs and symptoms must have been directly linked to a single agent

* We assigned each country to an African region ^†^ based on the United Nations Statistics Division (https://unstats.un.org/unsd/methodology/m49/).

^**†**^ Variable was used in the meta-regression models.

**Fig 2:**
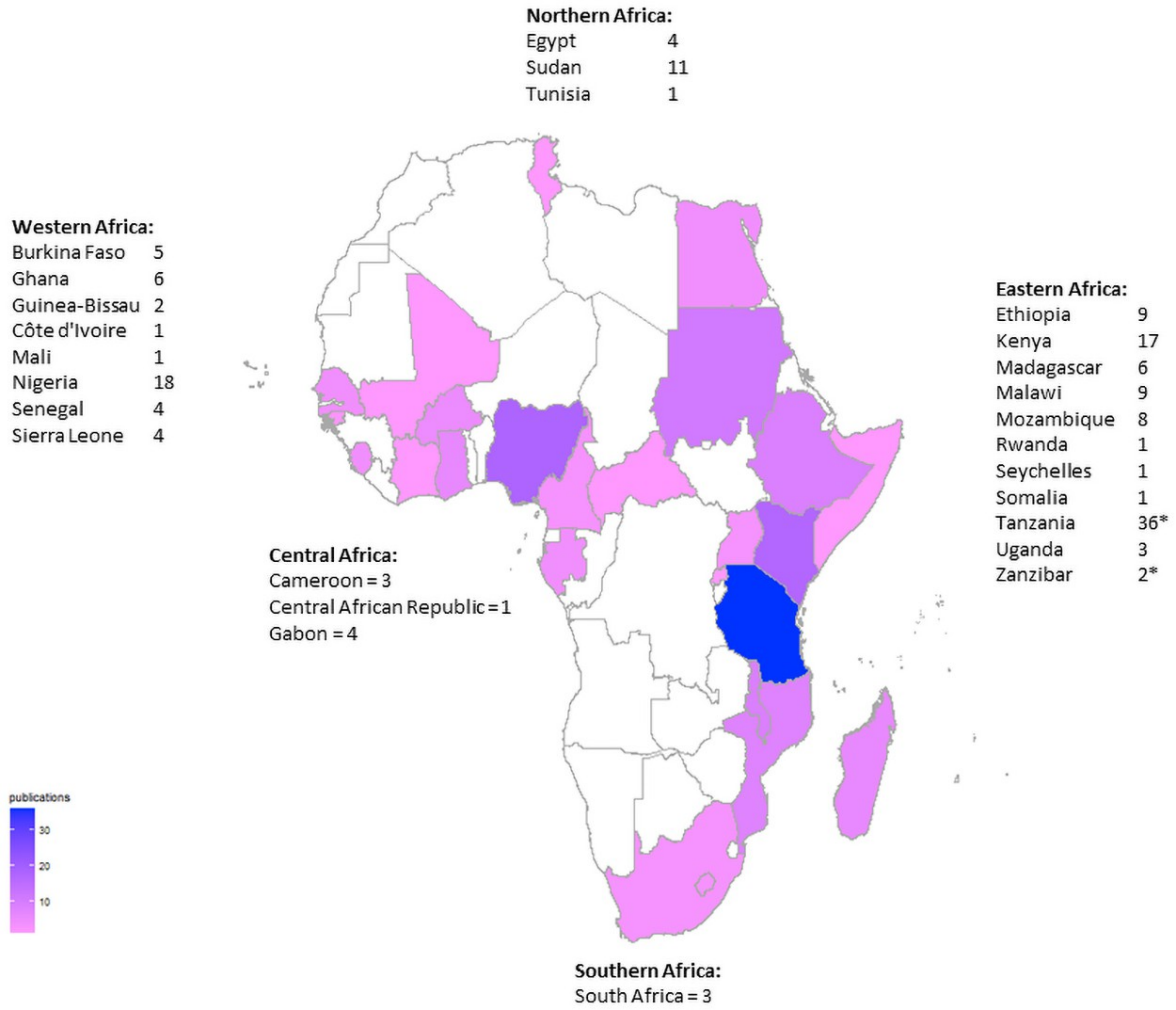
Number of studies per country included in meta-analysis of NMFIs in Africa. Studies on populations from multiple countries were treated as separate studies. *Tallies from Tanzania and Zanzibar were combined in the image (OpenStreetMap contributors, http://geoportal.icpac.net/layers/geonode%3Aafr_g2014_2013_0).

We adopted the following guidelines for extracting data:

(i.) Criteria related to agents. We did not extract data on co-occurring agents. We also did not include subsets of results if not all subsets within a group (such as species within a genus) had results presented. In such cases, the general results (such as for the genus) were extracted because they represented all the members within the group. Bacterial agents reported at levels higher than the genus (such as “coliforms”, “Enterobacteriaceae” or “Enterobacteria”) were not extracted. As some viruses (e.g., Adenoviruses) were commonly identified at levels higher than the genus, these were extracted and included in our tally of total agents (Figs 2 and 4 and Table 2). When agents were tested in more than one clinical sample (such as blood and urine), all these data were extracted. Data on infections diagnosed in blood were preferred in the total tally of agents in Africa as well as in the meta-analysis section. However, results from all samples tested were used in the total number of agents per sample tested.
(ii.) Criteria concerning the number of patients positively tested for an agent and the diagnostic test used. When more than one number of positive cases were reported due to more than one cut-off point used (e.g. PCR results for Parvovirus B19 and Parvovirus B19 >1000 IU/ml), the number presented by the authors in the final report was included in our analyses. When samples were pooled for laboratory analysis and results therefore not presented based on individual patient samples, we regarded the number of pools as the total study size. Data on the incidence of aetiologic agents were not extracted. Only the proportion of positive cases determined during the study period was extracted. When patients were recruited over several years in a cross-sectional study, and each patient was sampled only once, patients and positive cases reported during the study period were summed up in total and the proportion of positive cases was calculated. When screening and confirmatory tests were used, results from the confirmatory test were extracted. When different tests were used for patient management and subsequent treatment and others for the epidemiological study of the agent of interest, results from the latter were extracted. We also preferentially included results from IgG assays as we regarded them to be more inclusive in diagnosing both acute and convalescent cases in studies that utilised serological assays detecting IgM and IgG antibodies. When no diagnostic assay and/or only the total number of positive cases were recorded in a study, the agent was not included in the extracted dataset.
(iii.) Criteria concerning data on clinical signs and symptoms. We considered only data linked to a single agent of interest and not those reported for the entire study population when multiple agents were investigated. Additionally, we only extracted absolute numbers of agents linked to specific clinical signs and symptoms and not measures of association (such as odds ratios).
(iv.) Criteria concerning data on patient and study characteristics. When multiple countries were included in a study, data were extracted per country and the countries of the study populations described in the article were deemed as all the countries involved. In such cases, data on agents not presented per country were not extracted. When studies comprised febrile and non-febrile cohorts with data on agents presented separately, data from the febrile cohorts were extracted. When data were combined, the paper was rejected. When a study included HIV-positive and -negative patients and data were presented by these subgroups separately, data on HIV-negative patients were extracted to minimize bias related to opportunistic infections in immunocompromised patients. If this was not possible, data from both HIV-positive and -negative patients were extracted. We excluded data on febrile patients with diagnosed malignancies and on chemotherapy as immunocompromised states predispose these populations to infection. However, a separate study of common aetiological agents of non-malarial fevers in these populations would be more informative. When studies were based on the same population and agents reported more than once, albeit in different contexts (or not), we avoided extracting them more than once from the different publications. We assumed studies to be from the same population when expressly stated, or when dates and locations of sampling were the same. We confirmed this by checking any similarity of authors’ names. Lastly, we did not exclude any publications based on the age of study participants to be as inclusive as possible.

### Statistical analyses

Extracted data were entered into MS Excel and subsequently saved as individual comma-separated value files (.csv), imported, and merged in R statistical environment for analysis [25]. Bar charts were generated to present the percentage of fever cases testing positive for the different agents in the five African regions. Choropleth maps were created to show study countries on the continent. A heat map was produced to summarize clinical signs and symptoms. The samples tested for each agent were illustrated in chord diagrams (S2 Text). Boxplots were generated for various study characteristics and are presented in the supporting information (S4 Fig). Barplots for the quality assessment scores, the journal sources and publication years of included studies were also made (S1–S3 Figs). We also subdivided the publication years into those before or during the year 2010 and those after as the World Health Organisation (WHO) recommended the parasitological confirmation of all suspected malaria cases either by microscopy or rapid diagnostic tests in this year [26].

### Meta-analyses

PMr were calculated by dividing the number of diagnoses attributable to an aetiologic agent by the total number of diagnoses in a study [19]. Selected pathogens with high PMr estimates and more than ten records identified by our systematic review were selected for the meta-regression analyses. These included *Brucella* spp., Chikungunya virus, Dengue virus, *Haemophilus* spp., *Klebsiella* spp., *Leptospira* spp., *Salmonella* spp. (non-typhoidal and typhoidal), *Staphylococcus* spp., and *Streptococcus* spp. These pathogens served as indicator organisms in our review to investigate important determinants of PMr estimates. Random effects models were used to estimate the overall PMr and to quantify the heterogeneity (which refers to the amount of variation between the studies) evident for each outcome. The heterogeneity (Tau squared or τ^2^) was calculated using the restricted maximum-likelihood (REML) method. The proportion of this variation that was not attributable to chance was also given using Higgin’s *I*^2^ statistic [27]. A Hartung-Knapp adjustment was used in the models to account for the low study numbers and any possible heterogeneity between studies that was substantial [28,29]. Forest plots were generated with the studies on each agent arranged in chronological order and subgroups were analysed to separate study results according to the diagnostics applied for pathogen detection (direct, indirect, or both).

Meta-regression analyses were performed on the selected aetiologic agents to determine how the study and population characteristics influenced the summary estimates and contributed to the variation between studies. Only studies that had non-zero reports of diagnoses were included and analyses were initially run for each of the selected agents using the variables indicated in Table 1. Mixed-effects models were applied to each of these nine variables using the *metagen* command and the REML method as the τ^2^ estimator. Statistical significance was set at p value <0.1.

The following variables were included using a backward elimination approach into the final multivariable mixed-effects meta-regression models: population status, African region, study end date (year), recruitment place, and diagnostics. The study end date was deemed *a priori* to be a potential confounder and was therefore forced into all models. The τ^2^ was estimated using the REML method and a Hartung-Knapp adjustment was included as well. Only variables with p values <0.05 were regarded as statistically significant.

A factor analysis model of the quality assessment scores (risk of bias) was fitted to observe whether the results collectively reflected a single underlying latent variable that represents the "quality" of the studies included. The resulting eigenvalues did not support unidimensionality. Study quality scores were also analysed for each agent of interest using univariable analyses to identify questions that would be consistent predictors for all the agents, but none were found consistently significant. These scores were therefore not included in the meta-analyses any further.

## Results

A total of 5730 studies were identified by our database searches and 133 articles representing 391,835 febrile patients were finally included in the systematic review. These articles were in English or French.

The selection process is summarised in Fig 1 and the countries represented by the included studies are shown in Fig 2. A summary of study and population characteristics is given in S2 Table. The questions we answered to assess the risk of bias for each study along with graphical summaries of the quality assessment scores are also presented in the supporting information (S1 Fig). The number of included studies increased over time (S2 Fig), especially after 2010, suggesting a greater research interest and awareness on non-malarial fevers after the WHO recommendation for parasitological confirmation of suspected malaria cases.

The quality assessment revealed that sample size calculation was uncommon and non-probability sampling methods were frequently used. A total of 86% of the studies lacked detailed information on both. Consequently, the studies in our review may have suffered from selection bias in terms of inclusion criteria for patients. Descriptive statistics of study populations were consistently found. However, in data analysis, control of confounding factors was lacking in 87% of the studies. The proportion of positive cases and measures of association were reported with precision ranges (i.e., confidence intervals (CI)) in only half of the studies. There were few studies (14%) that analysed paired samples taken in the acute and the convalescent phase of disease. In general, the scores assigned to the introduction of study topics (including scientific background, objectives, setting, location, and date of the study as well as clinical data collection methods) and discussion of results (summarising the key findings, giving limitations, interpreting, and comparing results with other studies as well as stating the generalisability of the findings (external validity)) were high. While fever cases were often accurately defined, specifically by providing a minimum body temperature, the duration of fever, accompanying clinical signs and symptoms, and how cases were diagnosed, the location of temperature measurement was not stated in 57% of the studies.

Our review included a wide range of patient ages, with the median (interquartile range) of the minimum and maximum reported ages being 1.0 (0.2–25.0) and 35.5 (12.8–72.0) years. A summary of the measures of central tendency and spread given for the ages of study participants is given in S4 Fig.

We also assessed whether studies had considered both cases (e.g. febrile patients) and controls (e.g. afebrile patients or those lacking certain clinical manifestations depending on the illness in question). We found descriptions of control groups in seven studies all of which were unmatched [30–36].

**Fig 3:**
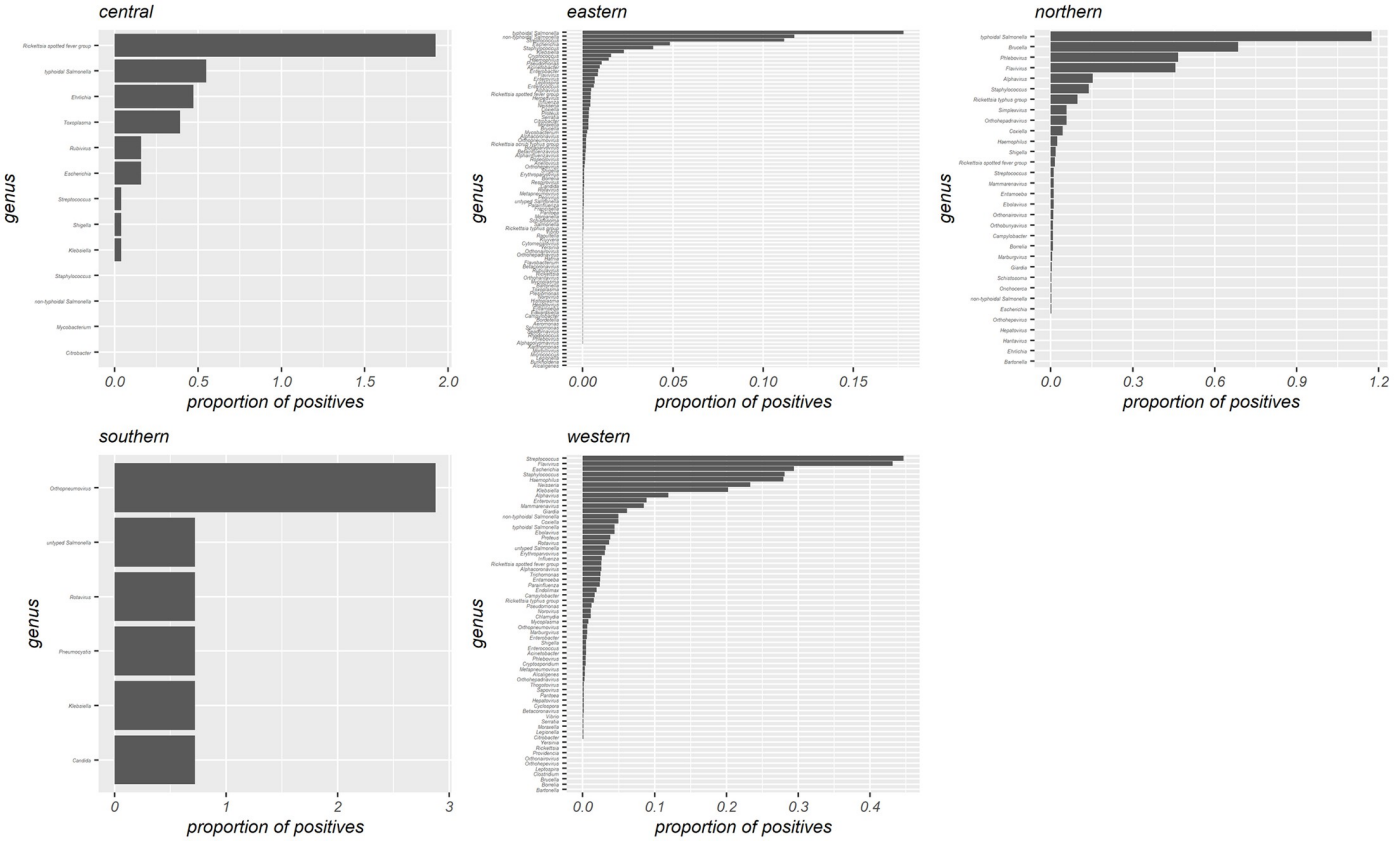
Aetiologic agents directly and/or indirectly detected in fever patients from different African regions. Proportions of positive cases were calculated by dividing the number of cases diagnosed by any kind of laboratory method by the total number of samples tested in each African region.

**Fig 4:**
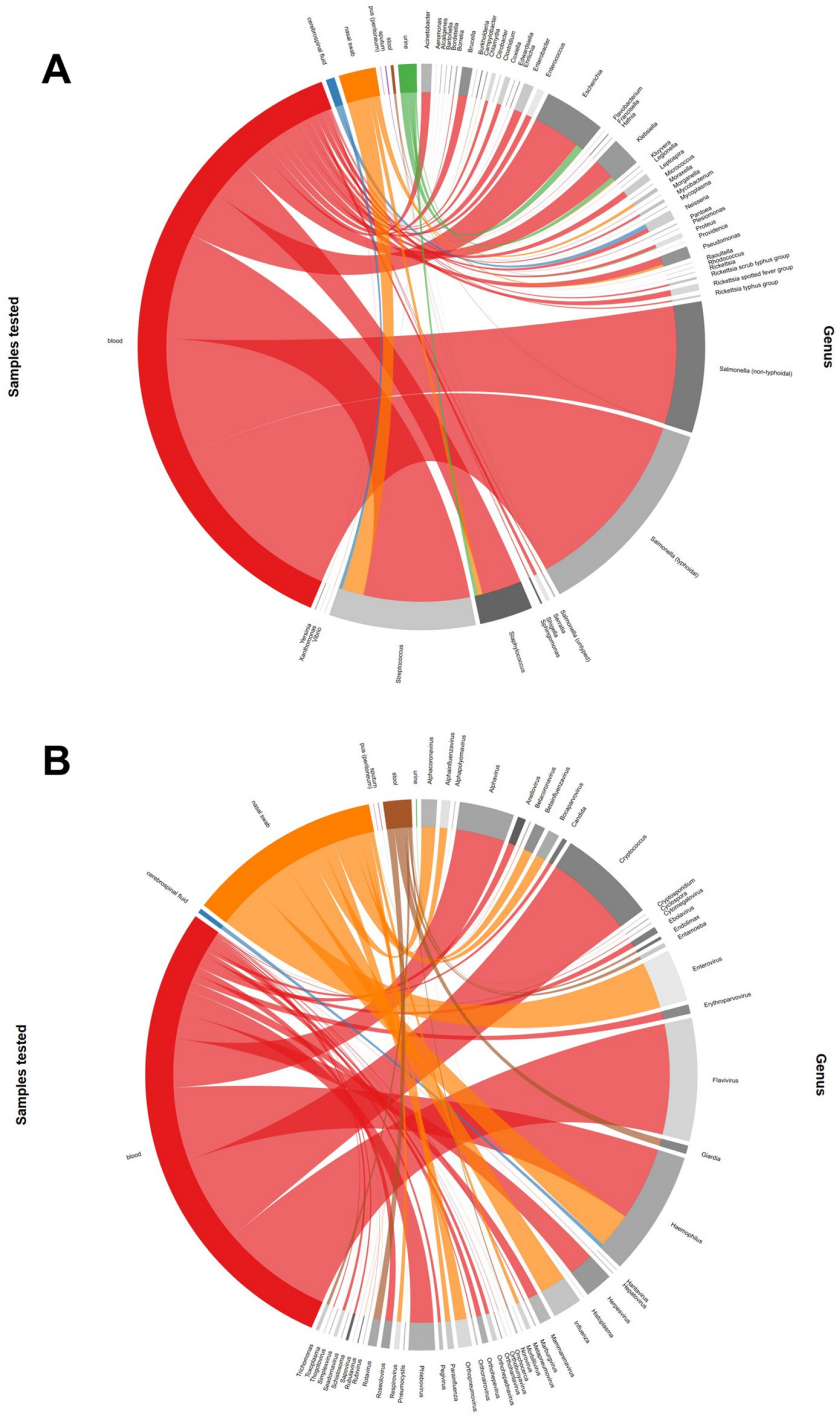
Chord diagrams presenting the samples tested for the diagnosis of (A) bacterial infections and (B) viral, parasitic, and fungal infections. Arc lengths represent the total numbers in each category. Blood samples comprised serum and plasma. Nasal swabs also included oro- and nasopharyngeal swabs and aspirates.

A wide array of aetiologic agents were identified to cause fever (Fig 3 and Table 2), with bacteria and viruses predominating in differing amounts depending on the geographical region. Chord diagrams relating the agents detected to the sample type are presented in Fig 4A (bacteria) and Fig 4B (viruses, parasites, and fungi).

**Table 2:** Most common aetiologic agents found in the reviewed literature to cause non-malarial febrile illnesses (NMFIs) based on number of publications.

Organism type	Aetiologic agent	References
**Bacteria**		
	*Staphylococcus* spp.	[37–81]
	*Streptococcus* spp.	[36,39,41–45,48–51,54–65,67,69,70,72–75,77,78,80–90]
	*Escherichia* spp.	[37,39,41–46,48–51,53–55,58–61,63–79,81,85,88,91–94]
	Typhoidal *Salmonella* spp.	[38,41,44,48,49,51,55,58,60–65,67,69,70,72,74,79,80,83,85,88,92,95–103]
	*Klebsiella* spp.	[37,39,42,43,45–50,55–61,66,68–73,75–77,81,83,93,104]
	Non-typhoidal *Salmonella* spp.	[41,44,48,49,51,55,57,58,60–65,67,69,70,72,75,77,80,85,87,88,92,100,105,106]
	*Pseudomonas* spp.	[41–43,45,46,48,49,55–59,62,69,72–75,77,80,83,93]
	*Haemophilus* spp.	[39,43,49,50,54–58,62,64,67,69,77,79,80,83,85,86,89,92,107]
	*Proteus* spp.	[37,42–44,46,47,49,53,59,66,69,71–77,93,96,99]
	*Leptospira* spp.	[33,49,56–58,82,98,108–115]
**Rickettsial organisms**		
	*Rickettsia* spotted fever group	[35,49,116–122]
	*Rickettsia* typhus group	[49,116–118,120–124]
	*Rickettsia* scrub typhus group	[49,116–118,120–124]
	Ungrouped *Rickettsia* spp.	[57,58,82]
	*Ehrlichia* spp.	[116,125]
**Viruses**		
	Flaviviruses	[32,34,49,56–58,87,97,114,122,126–138]
	Alphaviruses	[34,49,58,97,114,122,127,128,131,133,136,138–141]
	Phleboviruses	[34,49,111,122,127,136,142]
	Orthopneumoviruses	[36,49,56–58,104,111]
	Influenza viruses	[36,56–58,94,128,143]
	Rotaviruses	[49,58,63,104,128,144]
	Enteroviruses	[49,56–58,111,144]
	Alphacoronaviruses	[49,56–58,94]
	Orthohepadnaviruses	[58,111,122,127,144]
	Ebolaviruses	[34,87,94,122,136]
	Mammarenavirus	[30,34,122,136,145]
**Parasites and fungi**		
	*Cryptococcus* spp.	[41,44,48,55,69,84,85,106]
	*Candida* spp.	[41,44,69,72,74,78,104]
	*Entamoeba* spp.	[49,58,63,87]
	*Histoplasma* spp.	[48,84,85]
	*Giardia* spp.	[58,63,87]
	*Schistosoma* spp.	[122,146]
	*Toxoplasma* spp.	[49,95]
	*Pneumocystis* spp.	[104]
	*Cyclospora* spp.	[58]
	*Trichomonas* spp.	[63]
	*Cryptosporidium* spp.	[58]
	*Onchocerca* spp.	[122]
	*Endolimax* spp.	[63]

The largest number of agents were reported from the Eastern Africa region with typhoidal *Salmonella* as the leading cause of febrile illnesses. Western, Northern, Central, and Southern African regions followed with decreasing numbers of agents identified and the major agents being *Streptococcus* spp., typhoidal *Salmonella*, *Rickettsia* spp. spotted fever group, and orthopneumoviruses, respectively. Infections were either diagnosed from normally sterile samples, such as blood, urine, cerebrospinal fluid, or from nasal secretion, sputum, stool, and pus as presented in Fig 4. The most important bacterial agents found in blood were typhoidal and non-typhoidal *Salmonella*, *Streptococcus* spp., *Escherichia* spp., *Staphylococcus* spp., and *Klebsiella* spp. Viral agents diagnosed from blood samples were mostly flaviviruses and alphaviruses. In nasal swabs taken to prove upper respiratory tract infections, *Streptococcus* spp., enteroviruses, *Haemophilus* spp., *Staphylococcus* spp., influenza viruses, *Moraxella* spp., alphacoronaviruses, *Pseudomonas* spp., orthopneumoviruses, and bocaparvoviruses were most frequently found. Gastrointestinal infections diagnosed by stool samples were mostly caused by rotaviruses, *Giardia* spp., non-typhoidal *Salmonella*, *Entamoeba* spp., and *Trichomonas* spp. Urinary tract infections were typically diagnosed by the isolation of *Escherichia* spp., *Klebsiella* spp., and *Staphylococcus* spp. from urine samples. *Neisseria* spp., *Streptococcus* spp., *Haemophilus* spp. and *Klebsiella* spp. were responsible for infections of the central nervous system and were predominantly isolated from cerebrospinal fluid.

**Fig 5:**
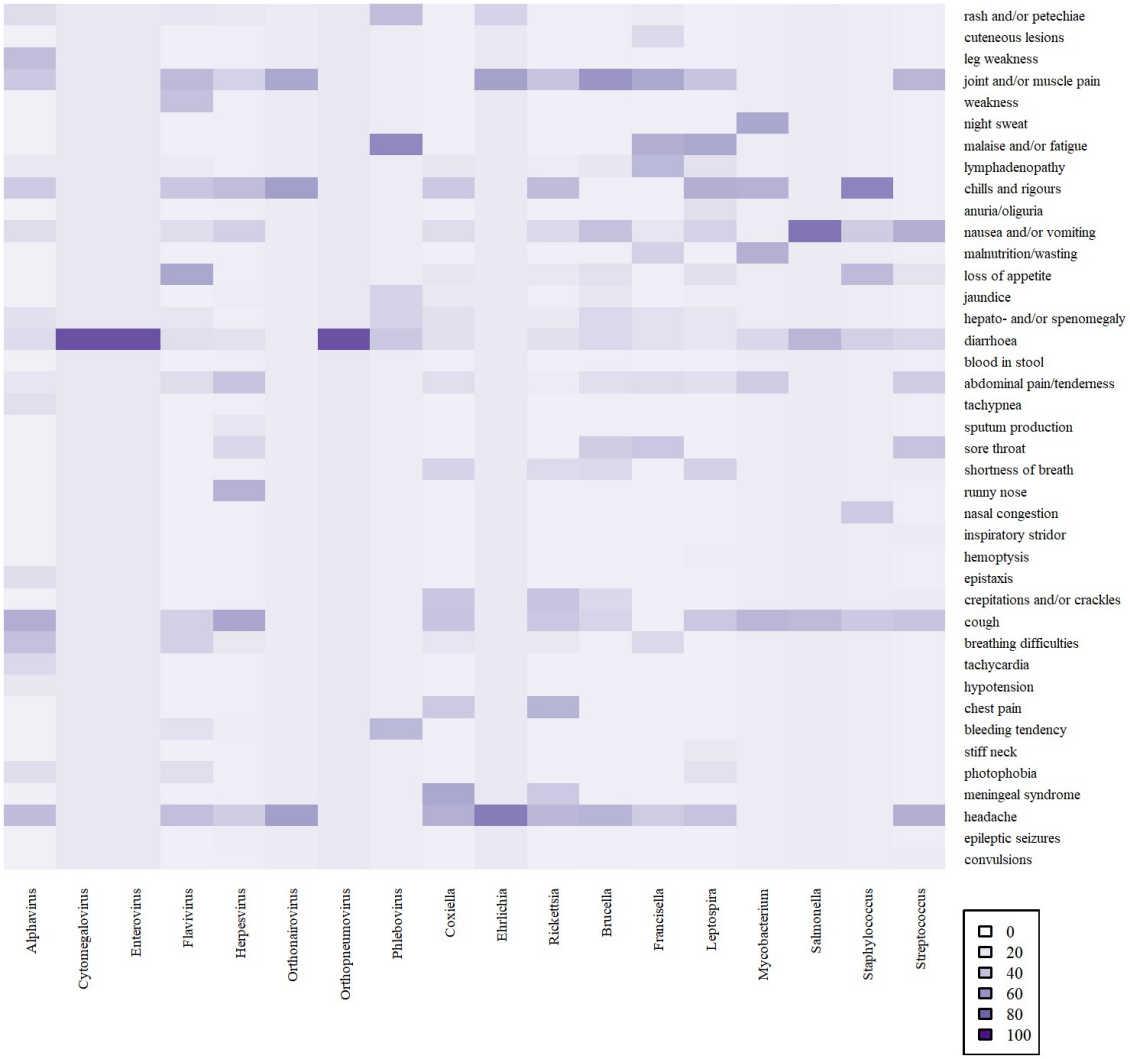
Heat map showing the occurrence of clinical signs and symptoms in patients infected with specific agents. (shading represents the proportion of positive cases (%)). Clinical data were only added to the heat map when linked to a particular pathogen.

Clinical signs and symptoms accompanying fever and associated with aetiologic agents were available from only 30 out of 130 articles included in our study. Their description was often scanty or vague and tended to be non-specific for a single infectious disease. Some of the studies had zero reports of clinical signs and symptoms associated with agents. A heat map relating agents to clinical signs and symptoms is presented in Fig 5.

Forest plots with sub-groups according to the type of diagnostics used to identify pathogenic agents are presented in the supporting information (S5–S14 Figs). Summary estimates of the selected agents were generally low, with the lowest being *Haemophilus* spp. (1.4%, 95% CI: 0.5–3.6) and the highest being Dengue virus (8.4%, 95% CI: 3.2–20.0). Significant heterogeneity was observed in all the analyses, with the least being for *Brucella* spp. (*I*^2^ = 94.6%, τ^2^ = 1.4) and the greatest being for *Haemophilus* spp. (*I*^2^ = 99.5%, τ^2^ = 5.3). Results of the summary and heterogeneity estimates as well as study characteristics and variables found significant in the meta-regression analyses are presented in Table 3. Further information on the results from multivariable analyses can be found in Table 4. The very high values of Higgin’s *I*^2^ reflect the fact that relative to the within-study variance, the between-study variance was really large. Results of the univariable meta-regression analyses are presented in the supporting information (S3 Table). In the final model, the diagnostic method was a significant predictor of PMr for typhoidal *Salmonella* (p value = 0.00) and Dengue (p value = 0.02). The population status was a significant predictor of PMr estimates for *Haemophilus* spp. (p value = 0.01). The study end date was a significant predictor of PMr estimates for *Klebsiella* spp. (p value = 0.04), and an increase in PMr estimates by 0.5% was observed with each additional year. The median end date of the studies on *Klebsiella* spp. was 2008 (interquartile range: 2000–2013). Two studies lacked a record of this date.

**Table 3:** Summary statistics of the studies investigating the agents included in meta-regression analyses.

Aetiologic agent	Number of studies investigating agent*	Study population sizes^†^		Overall heterogeneity	Summary estimates of PMr in % (95% CI) with the diagnostic tests used				Variables significant in the multivariable model^‡^
		Median	IQR		Overall	Direct	Indirect	Direct & indirect	
*Brucella* spp.	13	325	195–582	*I*^2^ = 94.6%, τ^2^ = 1.4	3.5% (1.7–7.1)	3.1% (0.9–10.5)	3.6% (1.2–10.5)	NA	None
Chikungunya virus	15	338	240–394	*I*^2^ = 98.8%, τ^2^ = 3.8	4.5% (1.5–12.7)	1.7% (0.2–11.5)	9.6% (2.7–29.2)	NA	None
Dengue virus	21	310	195–382	*I*^2^ = 98.9%, τ^2^ = 4.8	8.4% (3.2–20.0)	2.3% (0.6–7.9)	29.8% (13.8–53.0)	6.2% (0.0–100.0)	Diagnostics
*Haemophilus* spp.	23	522	341–1,711	*I*^2^ = 99.5%, τ^2^ = 5.3	1.4% (0.5–3.6)	1.4% (0.5–3.8)	1.6% (0.2–10.7)	NA	Population status
*Klebsiella* spp.	31	300	150–842	*I*^2^ = 98.9%, τ^2^ = 2.3	1.8% (1.0–3.1)	1.8% (1.0–3.2)	1.6% (0.2–10.7)	NA	Study end date
*Leptospira* spp.	15	223	180–379	*I*^2^ = 95.5%, τ^2^ = 4.2	3.2% (1.1–8.9)	0.5% (0.1–2.1)	9.6% (3.5–24.0)	22.9% (17.8–28.8)	None
Non-typhoidal *Salmonella* spp.	28	437	235–1,076	*I*^2^ = 99.5%, τ^2^ = 3.5	1.6% (0.8–3.3)	1.6% (0.8–3.3)	NA	NA	None
Typhoidal *Salmonella* spp.	34	449	243–1,156	*I*^2^ = 97.8%, τ^2^ = 1.8	2.0% (1.3–3.1)	1.4% (0.9–2.3)	8.5% (4.0–17.4)	3.4% (0.3–32.4)	Diagnostics
*Staphylococcus* spp.	45	284	170–638	*I*^2^ = 98.7%, τ^2^ = 2.1	2.1% (1.4–3.3)	2.1% (1.4–3.3)	1.6% (0.2–10.7)	NA	None
*Streptococcus* spp.	43	277	119–636	*I*^2^ = 99.1%, τ^2^ = 2.9	3.2% (2.0–5.3)	3.2% (1.9–5.3)	4.9% (1.6–14.2)	NA	None

CI; confidence intervals, IQR; interquartile range, NA; not available

* We may have subdivided study populations during the analyses

^**†**^ Study population size refers to the total number of samples tested

^**‡**^ Results of the final multivariable meta-regression models are expounded in Table 4

**Table 4:** Results of the final multivariable meta-regression models of aetiologic agents. Statistical significance was set at p value <0.05.

Variables*	Coefficient^†^	Standard error	p value	95% confidence intervals of coefficient	
				lower	upper
Dengue					
Intercept**	0.07	0.07	0.29	-0.07	0.22
Study end date (centred)^‡^	-0.00	0.00	0.37	-0.01	0.00
Diagnostics (p value = 0.02)					
direct and indirect	0.22	0.16	0.19	-0.12	0.57
indirect	0.29	0.10	0.01	0.09	0.50
*Haemophilus* spp.					
Intercept^§^^,^ **	-0.01	0.05	0.87	-0.11	0.09
Population status (p value = 0.01)					
inpatient/outpatient	0.29	0.09	0.00	0.12	0.50
outpatient	0.02	0.12	0.86	-0.23	0.27
Study end date (centred)^‡^	0.01	0.00	0.08	-0.00	0.01
*Klebsiella* spp.					
Intercept**	0.08	0.02	0.01	0.02	0.13
Population status (p value = 0.81)					
inpatient/outpatient	0.02	0.04	0.63	-0.07	0.11
outpatient	-0.01	0.04	0.81	-0.08	0.07
Study end date (centred)^‡^	-0.00	0.00	0.04	-0.01	-0.00
Typhoidal *Salmonella* spp.					
Intercept**	0.03	0.01	0.00	0.01	0.04
Study end date (centred)^‡^	-0.00	0.00	0.67	-0.00	0.00
Diagnostics (p value = 0.00)					
direct and indirect	0.01	0.02	0.58	-0.03	0.06
indirect	0.07	0.02	0.00	0.03	0.10

* The overall p value of the variable is written next to the variable heading. Redundant variables were dropped from the model.

^**†**^ When exponentiated, the coefficient gives the odds ratio (OR) of the effect of the variable to the proportion of positive cases.

^**‡**^ The study end date was centred at the year 2000. Therefore, the intercept refers to a study conducted in 2000. This variable was also regarded *a priori* to be a potential confounder and therefore forced into the models.

^**§**^ The intercept was a negative value in *Haemophilus* spp. as a result of the influence from two outlying studies.

** The intercept represents a baseline individual, i.e. an individual with values of zero for all variables in the model.

## Discussion

We reviewed the literature on the various NMFIs in Africa and summarised their occurrence in the five African regions. Three critical results were generated: (i) common aetiological agents of non-malarial fevers in Africa, (ii) summary effects for selected agents from meta-analyses and, (iii) important predictors of the proportional morbidity rates for these agents from meta-regression analyses. Findings from this review will inform the prioritisation of non-malarial febrile illnesses in Africa and provide important parameters to be carefully considered in future fever studies on the continent. Although we applied lenient inclusion criteria, only a few studies turned out to be eligible for our systematic review. The 133 studies finally included covered 25 out of 54 UN member states in Africa. The majority of studies were conducted in Eastern Africa, and other African regions were underrepresented. This underrepresentation stands out to particularly populous countries such as the Democratic Republic of Congo (DRC). Investment in research on these illnesses in such countries will greatly improve our understanding of disease burden, especially in sub-Saharan Africa. The exposure of human populations to several NMFI agents has been demonstrated in DRC [147–149]. This highlights the need for more collaborative research in African regions, especially outside Eastern Africa to gain deeper insights into the epidemiology of NMFIs [150]. The high number of publications in the Eastern Africa region could be explained by growing research interest in zoonoses. Recent reviews have highlighted a large number of foreign-led research projects on zoonoses in the Horn of Africa region [151,152]. We also observed generally more studies being performed after 2010 than the period before. This could have resulted from the more readily available malaria diagnostics which improved differential diagnosis of febrile illnesses on the continent, resulting in increased awareness of NMFI in the public health community. Among the included studies, our quality assessment revealed many studies being weak in patient selection methods, or in giving descriptions thereof. Data analysis methods were also found to be a potential source of bias. Therefore, we recommend adequate sample size calculations in future studies on NMFIs in Africa to enable the selection of statistically representative populations. We included studies investigating patients of any age in our review to be more inclusive. Despite several publications mentioning the age of study participants, many did not stratify the occurrence of the various agents by age groups. In the few that stratified, different age groups were used, making it difficult to summarise information on various agents with different age groups. We also found the age variable more often described by measures of central tendency and dispersion than being categorised into age groups. Consequently, we did not analyse the occurrence of agents in different ages, despite there being likely important differences in leading agents of non-malarial fevers in children and adults. For instance, acute respiratory infections have been demonstrated as leading causes of febrile illness in children under five years [36,49]. However, there exists a rising number of standardised protocols from large multi-centre studies investigating non-malarial fevers which offer an opportunity to harmonise this important source of study heterogeneity in Africa [101,105,153–155]. Only a few studies included both cases and controls in the study design. Estimating the burden of non-malarial fevers in febrile patients can be confounded when the illness is endemic. The detection of these pathogens in febrile patients may therefore not necessarily mean clinical significance. Hence, the inclusion of control groups (e.g. afebrile patients) can help to minimise the chances of incidental findings. Given that only a few studies used such controls, it must be assumed that the pathogens diagnosed played a role in the febrile illnesses. The summary estimates for Dengue and Chikungunya viruses were comparable to what other meta-analyses had estimated among febrile patients in Africa [156,157]. Lower summary estimates for *H*. *influenzae* associated with bacterial meningitis were found [158] but estimates for febrile patients were not available. We also could not identify any summary estimates for *Brucella* spp., *Klebsiella* spp., *Leptospira* spp., typhoidal and non-typhoidal *Salmonella* spp., *Staphylococcus* spp., and *Streptococcus* spp. among febrile populations. Overall, we observed low summary estimates for most agents in our meta-analyses, probably resulting from the use of diagnostic tests with low sensitivity. The high levels of heterogeneity we observed for all agents likely arose from the variety of diagnostic methods in use on the continent [159].

We identified only a few protozoal and helminthic causes of fever in a total of seven studies. The disproportionate burden of some of these agents in HIV-positive patients [160,161] and their contribution to diarrhoeal diseases [162] should warrant their inclusion in future fever studies to better understand their public health impact. As we excluded results from HIV-positive patients in our review, we might have eliminated HIV-associated agents. Many of the studies in our review either had patients not tested for HIV or the study results not analysed separately according to HIV status, making it likely we inadvertently included HIV-positive patients in our analysis. HIV counselling and testing is recommended for the management of febrile illnesses in countries with endemic HIV transmission among adults and adolescents, due to the strong association of certain agents with an HIV-positive status [163]. Hence, there is a need for most African countries to adopt standardized protocols for the management of febrile patients so that important aetiologic agents are not overlooked, comparable to existing protocols for adults and adolescents in malaria-endemic countries [163].

The commonly used microbiological methods for diagnosing the different aetiologic agents varied considerably. The choice of diagnostic test was a significant predictor for typhoidal *Salmonella* spp. in the multivariable model. Culture and isolation are known to be the gold standard for the diagnosis of enteric fever [164] and allow for pathogen characterisation, which is important in understanding the epidemiology of bacterial infections. However, this approach is expensive, laborious, and time-consuming. In resource-scarce settings, serological diagnosis of infections from typhoidal *Salmonella* is therefore indispensable. Although these methods are suitable, a gold standard against which other serological tests can be compared is missing. Due to the large variety of tests and cut-offs in different populations, as well as cross-reactivity with other bacteria, results from serological tests are often inconsistent. Consequently, diagnostic methods contribute to the variation between studies in Africa and the high heterogeneity values observed. Similarly, the choice of diagnostic test was a significant predictor for Dengue PMr. A wide range of indirect detection methods such as neutralization, IgM and IgG ELISA and lateral flow assays were utilised in the included studies because of their lower costs as well as their reasonable sensitivity and specificity. Serological results however have to be interpreted with caution, carefully considering the timing of serum collection and the chance for following up a patient by paired sample testing in both the acute and the convalescent phase of infection [165]. A combination of indirect and direct detection methods could work well in harmonising diagnostic results across the continent. The establishment of tiered laboratory systems has been proposed in endemic countries to improve screening and confirmatory testing [166]. In the case of Dengue as well, variation in diagnostics may have contributed largely to the differences between studies. Because of the high heterogeneity observed, we did not perform publication bias analyses for any of the agents [159].

Population status was a significant predictor for *Haemophilus* spp. in the multivariable model. The majority of studies focussed on inpatients. However, studies that included both inpatients and outpatients identified *Haemophilus* spp. more frequently than those investigating outpatients only. This indicates that *Haemophilus* spp. were mainly identified after fever patients had been admitted to hospital. Therefore, primary intervention strategies such as *Haemophilus influenzae* type B (Hib) vaccination at healthcare centres, which has been linked to reduced numbers of infections in several African countries [167], are a viable option. In addition, there is a need for monitoring antibiotic resistance in *H*. *influenzae* and *H*. *parainfluenzae* isolates to ensure effective treatment for febrile patients. Despite antibiotic resistance being reported in some studies, ciprofloxacin has been shown to be effective in treating bacteraemia associated with *Haemophilus* spp. and suitable for outpatients in Zanzibar [79].

The proportion of *Klebsiella* spp. cases was significantly associated with the study period. Each additional year increased the PMr estimates by 0.5%. *Klebsiella pneumoniae* is one of the Enterobacteriaceae classified as a high priority pathogen (critical) for research and development due to its increased resistance to carbapenems and third-generation cephalosporins [168]. Infections from these extended-spectrum beta-lactamase (ESBL)-producing *Klebsiella* spp. could lead to limited treatment options as well as treatment failures. This public health hazard may have been a reason for growing research interest in Africa over time. However, drivers of antibiotic resistance such as the misuse of antimicrobials in hospitals and agriculture may have simply increased the number of infections from these pathogens [169]. Harmonisation of antimicrobial resistance (AMR) surveillance in priority bacteria is highly recommended across the African continent and on a global scale to effectively control infections difficult to treat [170,171].

Our systematic review had several limitations. (I) Since only a few studies were identified, our analyses may have lacked statistical power to detect factors affecting the PMr estimates of the many aetiologic agents under study. (II) Several agents important for Africa were not included in the meta-analyses because we deemed the number of corresponding studies too low. These comprised mycobacteria, rickettsia, protozoa, and helminths. However, the indicator agents included in our meta-analyses can inform the design of future studies. (III) The wide variety of diagnostic methods to identify the causative agents of febrile illnesses were potential sources of information bias in our review. We, therefore, merged diagnostic results at the genus level in most cases. However, the knowledge of infective species is still essential for refining pathogen-specific control measures. (IV) PMr might have been overestimated if multiple agents were assigned to the diagnosis of an individual patient. This may happen in studies investigating more than one agent in a population. However, single aetiology studies were less frequent (S2 Fig). (V) As we targeted HIV-negative patients in our review, we may have also included those with unknown HIV results since the majority of studies did not test for HIV or did not report on the HIV status of febrile patients. Additionally, some African countries such as those in Eastern and Southern Africa still have a high HIV/AIDS incidence, even though there has been a 43% reduction in new cases in the last 10 years largely because of the rollout of antiretroviral therapy by national HIV control programmes. [172,173]. Excluding HIV patients from our review could have left out a considerable amount of NMFI cases, because it is very likely that many people with acute HIV infection may seek care for febrile illnesses [5,174]. There still exists little data on the interaction between HIV and several febrile illnesses, but some opportunistic bacterial and fungal infections are disproportionately associated with HIV-positive patients [41,48,69,85,175]. Therefore, the strength of association of various emerging NMFIs and HIV is an important consideration for future fever studies on the continent.

In conclusion, NMFIs are quite common in fever patients in Africa. With the current COVID-19 pandemic, control of NMFIs has been impeded because (I) clinical presentations tend to overlap, (II) access to healthcare services is restricted as a result of physical distancing measures and the extra demand for personal protective equipment, and (III) the capacity of strained health systems is insufficient [176]. In addition, the prioritisation of COVID-19 by public health authorities could cause diagnostic and therapeutic delays for NMFIs leading to a higher disease burden. However, there is an opportunity to integrate locally endemic NMFIs in the control of pandemic diseases such as COVID-19.

To reduce the huge variation observed between available fever studies, harmonisation of methodology is needful, with particular respect to case definition, study design (considering sample size estimations to minimise selection bias), and use of diagnostic methods. This will help improve summary estimates in future meta-analyses for aetiologic agents of NMFIs and may also encourage more similar fever studies across the continent in order to reduce the regional bias observed in our review. Previously present public health threats such as endemic infectious diseases (especially malaria, HIV/AIDS, and tuberculosis), non-communicable diseases, and accidents and injuries are being compounded by emerging and re-emerging infectious agents. Surveillance programs focusing on individual threats have been effective so far. However, with rising threats in resource-scarce settings, integrating disease surveillance and response systems can offer better public health outcomes [177]. Efforts led by the African Union to coordinate regional public health surveillance through the African Centres for Disease Control (Africa CDC) established in 2016 offer a great opportunity to strengthen and support African countries’ public health systems in surveillance, improving diagnostic capacity and promoting other public health practices in keeping with international targets such as the Sustainable Development Goals [6]. As these concerted efforts are already in motion for emerging pathogens such as SARS-CoV-2, ebola viruses and AMR bacteria, a good chance for a coordinated response to NMFIs exists.

## Supporting information

S1 Table Summary of the search terms applied, to retrieve the literature for our systematic review from different databases. (DOCX)

S2 Table Summary of the characteristics of studies included in the systematic review and subsequent meta-analysis. (DOCX)

S3 Table Results (p values) of univariable meta-regression analyses. (DOCX)

S1 Text Risk of bias assessment tool. (DOCX)

S2 Text Statistical analyses. (DOCX)

S1 PRISMA Checklist PRISMA 2020 Checklist. (DOCX)

S1 Fig Distribution of the risk of bias scores for the studies included in our systematic review and subsequent meta-analysis. (DOCX)

S2 Fig Distribution of included studies by publication year and type of study (whether more than one genus was investigated [multiple aetiologies study] or not [single aetiology study]). The demarcating line on the x-axis (in blue) shows publication numbers before and after 2010 when the World Health Organisation recommended parasitological confirmation of suspected malaria cases (either through microscopy or rapid diagnostic tests).
(DOCX)

S3 Fig Journal sources of the included studies. (DOCX)

S4 Fig Characteristics of the included studies and the populations represented. These also represent the variables we created and used in the meta-regression analyses **A**. Age (in years) distributions in the study populations **B**. Minimum temperatures (in °C) measured in fever patients at different locations of the body **C**. Duration of fever (categories of 1 to 24 hrs up to 2 weeks) in the study populations (sample size) **D**. Status and number (sample size) of the recruited study participants **E**. Recruitment places of study participants (sample size) **F**. Study populations (sample size) recruited in different settings.
(DOCX)

S5 Fig Forest plot of studies investigating typhoidal *Salmonella* (mostly comprised of *Salmonella* Typhi and *S*. Paratyphi) in order of increasing study year (referring to the end of sample collection). The summary estimate for typhoidal *Salmonella* among 293,981 patients tested was 2.0% (95% CI: 1.3–3.1). Between-study heterogeneity was significantly high (*I*^2^ = 97.8%, τ^2^ = 1.8).
(DOCX)

S6 Fig Forest plot of studies investigating non-typhoidal *Salmonella* (with identified serovars including Typhimurium, Enteritidis, Dublin, Infantis) in order of increasing study end year. The summary estimate for non-typhoidal *Salmonella* among 292,792 patients tested was 1.6% (95% CI. 0.8–3.3). Between-study heterogeneity was significantly high (*I*^2^ = 99.5%, τ^2^ = 3.5).
(DOCX)

S7 Fig Forest plot of studies investigating Dengue virus presented by increasing study end year (Adedayo et al. lacked study end date). The summary estimate for Dengue virus among 20,112 patients tested was 8.4% (95% CI: 3.2–20.0). Between-study heterogeneity was significantly high (*I*^2^ = 98.9%, τ^2^ = 4.8).
(DOCX)

S8 Fig Forest plot of studies investigating Chikungunya virus presented by increasing study end year. The summary estimate for Chikungunya virus among 18,080 patients tested was 4.5% (95% CI: 1.5–12.7). Between-study heterogeneity was significantly high (*I*^2^ = 98.8%, τ^2^ = 3.8).
(DOCX)

S9 Fig Forest plot of studies investigating *Haemophilus* spp. (with identified species including *H*. *influenzae* and *H*. *parainfluenzae*) presented by increasing study end year. The summary estimate for *Haemophilus* spp. among 240,446 patients tested was 1.4% (95% CI: 0.5–3.6). Between-study heterogeneity was significantly high (*I*^2^ = 99.5%, τ^2^ = 5.3).
(DOCX)

S10 Fig Forest plot of studies investigating *Staphylococcus* spp. (with identified species including *S*. *aureus* and *S*. *epidermidis*) presented by increasing study end year (Adjei et al. lacked study end date). The summary estimate for *Staphylococcus* spp. among 250,500 patients tested was 2.1% (95% CI: 1.4–3.3). Between-study heterogeneity was significantly high (*I*^2^ = 98.7%, τ^2^ = 2.1).
(DOCX)

S11 Fig Forest plot of studies investigating *Streptococcus* spp. presented by increasing study end year. The summary estimate for *Streptococcus* spp. among 241,560 patients tested was 3.2% (95% CI: 2.0–5.3). Between-study heterogeneity was significantly high (*I*^2^ = 99.1%, τ^2^ = 2.9).
(DOCX)

S12 Fig Forest plot of studies investigating *Leptospira* spp. presented by increasing study end year. The summary estimate for *Leptospira* spp. among 7,182 patients tested was 3.2% (95% CI: 1.1–8.9). Between-study heterogeneity was significantly high (*I*^2^ = 95.5%, τ^2^ = 4.2).
(DOCX)

S13 Fig Forest plot of studies investigating *Brucella* spp. presented by increasing study end year (Mustafa et al. lacked study end date). The summary estimate for *Brucella* spp. among 16,717 patients tested was 3.5% (95% CI: 1.7–7.1). Between-study heterogeneity was significantly high (*I*^2^ = 94.6%, τ^2^ = 1.4).
(DOCX)

S14 Fig Forest plot of studies investigating *Klebsiella* spp. presented by increasing study end year (Adjei et al. lacked study end date). The summary estimate for *Klebsiella* spp. among 226,762 patients tested was 1.8% (95% CI: 1.0–3.1). Between-study heterogeneity was significantly high (*I*^2^ = 98.9%, τ^2^ = 2.3).
(DOCX)
