# Supplementary material for: A systematic review and meta-analysis of the aetiological agents of non-malarial febrile illnesses in Africa
Source: PLoS Negl Trop Dis. 2022 Jan 24;16(1):e0010144. doi: 10.1371/journal.pntd.0010144 (PMC8812962; doi:10.1371/journal.pntd.0010144)
Supplement: S1 Table — (DOCX) [file pntd.0010144.s001.docx]

# Search strategy

## S1 Table: Summary of the search terms applied, to retrieve the literature for our systematic review from different databases.

| **Database** | **Search terms** | **Search conditions** | **Time span (publication year) of the search results** |
| --- | --- | --- | --- |
| **African Journals Online (AJOL)** | (undifferentiated OR unknown OR non-malaria OR non-malarial OR nonmalaria OR nonmalarial OR 'non-malaria' OR 'non-malarial') AND ('fever' OR 'pyrexia' OR 'hyperthermia' OR 'febrile') AND 'Africa' | Through Google search (site:www.ajol.info) | 1955 - 2019 |
| **Embase** | ('undifferentiated' OR 'unknown' OR 'non*malaria*') AND ('fever' OR 'pyrexia' OR 'hyperthermia' OR 'febrile') AND 'Africa' |  | 1973 - 2019 |
| **PubMed** | ("undifferentiated" OR "unknown" OR "non*malaria*") AND ("fever" OR "pyrexia" OR "hyperthermia" OR "febrile") AND Africa | Included both MeSH terms and plain words | 1969 - 2018 |
| **Scopus** | (ALL ((“undifferentiated" OR "unknown" OR "non*malaria*")) AND ALL (("fever" OR "pyrexia" OR "hyperthermia" OR "febrile")) AND ALL (Africa)) |  | 1930 - 2019 |
| **Web of Science** | ALL= ("undifferentiated" OR "unknown" OR "non*malaria*") AND ALL= ("fever" OR "pyrexia" OR "hyperthermia" OR "febrile") AND ALL= ("Africa") | Indexes=SCI-EXPANDED, CPCI-S, CPCI-SSH, BKCI-S, BKCI-SSH, CCR-EXPANDED, IC Timespan=All years | 1981 - 2018 |
