# Supplementary material for: A systematic review and meta-analysis of the aetiological agents of non-malarial febrile illnesses in Africa
Source: PLoS Negl Trop Dis. 2022 Jan 24;16(1):e0010144. doi: 10.1371/journal.pntd.0010144 (PMC8812962; doi:10.1371/journal.pntd.0010144)
Supplement: S2 Table — (DOCX) [file pntd.0010144.s002.docx]

# Summary of study characteristics

## S2 Table: Summary of the characteristics of studies included in the systematic review and subsequent meta-analysis.

|  | **First author** | **Aetiologies investigated** | **Country*** | **African region** | **Start year** | **End year** | **Study type** | **Study setting** | **Recruitment place** | **Population status** | **Sample**  **size**† | **Case definition^‡^** |
| --- | --- | --- | --- | --- | --- | --- | --- | --- | --- | --- | --- | --- |
| [1] | Aarsland | multiple | Ethiopia | Eastern | 2009 | 2010 | cross-sectional | urban | hospital | outpatient | 102 | axillary (38.1) |
| [2] | Achonduh-Atijegbe | multiple | Cameroon | Central | 2014 | 2014 | cross-sectional | urban | hospital | outpatient | 315 | axillary (38), 24h |
| [3] | Adam | single | Sudan | Northern | 2012 | 2013 | cross-sectional | NA | hospital | outpatient | 379 | NA |
| [4] | Adedayo | single | Nigeria | Western | NA | NA | cross-sectional | urban | hospital | NA | 130 | NA |
| [5] | Adjei | multiple | Ghana | Western | NA | NA | cross-sectional | urban | hospital | inpatient | 150 | NA |
| [6] | Afifi | multiple | Egypt | Northern | 1999 | 2003 | cross-sectional | urban | hospital | inpatient | 9,883 | oral (38.5), rectal (38.5), >2d |
| [7] | Akhuemokhan | single | Nigeria | Western | 2009 | 2010 | cross-sectional | urban/rural | hospital | inpatient | 373 | no location (38), 8h |
| [8] | Akpede**^§^** | multiple | Nigeria | Western | 1988 | 1989 | cross-sectional | urban | hospital | inpatient | 642 | rectal (40), <7d |
| [9] | Akpede**^§^** | multiple | Nigeria | Western | 1988 | 1989 | cross-sectional | urban | hospital | inpatient | 522 | ≤7d |
| [10] | Akpede**^§^** | multiple | Nigeria | Western | 1988 | 1989 | cross-sectional | urban | hospital | NA | 642 | rectal (38) |
| [11] | Animut | multiple | Ethiopia | Eastern | 2006 | 2006 | cross-sectional | rural | hospital/primary healthcare facility | outpatient | 653 | no location (37.5), 3d |
| [12] | António | single | Mozambique | Eastern | 2015 | 2016 | cross-sectional | urban | hospital/primary healthcare facility | outpatient | 392 | NA |
| [13] | Archibald | multiple | Tanzania | Eastern | 1995 | 1995 | cross-sectional | urban | hospital | inpatient | 235 | axillary (37.6) |
| [14] | Archibald | multiple | Malawi | Eastern | 1997 | 1997 | cross-sectional | urban | hospital | inpatient/outpatient | 173 | axillary (37.5) |
| [15] | Ayoola | multiple | Nigeria | Western | 1998 | 1998 | cross-sectional | urban | hospital | inpatient | 102 | rectal (38) |
| [16] | Ba | multiple | Senegal | Western | 2006 | 2008 | surveillance | urban/rural | hospital | NA | 1,711 | axillary (38), rectal (38.5) |
| [17] | Baba | multiple | Nigeria | Western | 2008 | 2008 | cross-sectional | urban | hospital | outpatient | 310 | no location (39) |
| [18] | Baudin | multiple | Sudan | Northern | 2011 | 2012 | cross-sectional | NA | hospital | NA | 130 | NA |
| [19] | Bell | multiple | Malawi | Eastern | 1988 | 1988 | cross-sectional | urban | hospital | inpatient/outpatient | 238 | axillary (37.5), 12h |
| [20] | Bevilacqua | single | Tanzania | Eastern | 2007 | 2007 | cross-sectional | rural | hospital | outpatient | 297 | no location (38), 10d |
| [21] | Biggs**^¶^** | single | Tanzania | Eastern | 2007 | 2008 | cross-sectional/paired samples | urban/rural | hospital | inpatient | 870 | axillary (37.5), oral (38), rectal (38), 48h |
| [22] | Biscornet | single | Seychelles | Eastern | 2014 | 2015 | cross-sectional | urban/rural | hospital | inpatient/outpatient | 223 | no location (38), 3d |
| [23] | Boone**^\|\|^** | multiple | Madagascar | Eastern | 2011 | 2013 | cross-sectional | urban/rural | primary healthcare facility | inpatient/outpatient | 1,020 | no location (38.5) |
| [24] | Botros | single | Egypt | Northern | 1983 | 1989 | cross-sectional | rural | hospital | outpatient | 50 | NA |
| [25] | Bouley**^¶^** | multiple | Tanzania | Eastern | 2007 | 2008 | cross-sectional | urban/rural | hospital | inpatient | 208 | axillary (37.5), oral (38), rectal (37.5) |
| [26] | Bouyou-Akotet | multiple | Gabon | Central | 2008 | 2008 | cross-sectional | urban | hospital | inpatient/outpatient | 418 | rectal (38), 24h |
| [27] | Brown | multiple | Nigeria | Western | 1999 | 2000 | cross-sectional | urban | hospital | NA | 37 | no location (37.5) |
| [28] | Brown | single | Somalia | Eastern | 1986 | 1986 | cross-sectional | rural | hospital | outpatient | 134 | no location (38) |
| [29] | Camara | single | Tanzania | Eastern | 2014 | 2014 | cross-sectional | NA | hospital/primary healthcare facility | NA | 483 | axillary (38), 2-9d |
| [30] | Cash-Goldwasser^**^ | single | Tanzania | Eastern | 2012 | 2014 | cross-sectional/paired samples | urban/rural/peri-urban | hospital | inpatient/outpatient | 562 | axillary (37.5), oral (38), rectal (38), tympanic (38), 72h |
| [31] | Chau | single | Mozambique | Eastern | 2012 | 2014 | cross-sectional/paired samples | suburban | primary healthcare facility | outpatient | 200 | axillary (37.6) |
| [32] | Chipwaza**^††^** | multiple | Tanzania | Eastern | 2013 | 2013 | cross-sectional | NA | hospital | outpatient | 370 | axillary (37.5), rectal (38) |
| [33] | Chipwaza**^††^** | multiple | Tanzania | Eastern | 2013 | 2013 | cross-sectional | NA | hospital | outpatient | 364 | axillary (37.5), rectal (38) |
| [34] | Christopher | multiple | Tanzania | Eastern | 2011 | 2012 | cross-sectional | NA | hospital | inpatient | 317 | axillary (37.5) |
| [35] | Commey | multiple | Ghana | Western | 1988 | 1989 | cross-sectional | urban | hospital | inpatient | 33 | NA |
| [36] | Crump**^¶^** | multiple | Tanzania | Eastern | 2007 | 2008 | cross-sectional | urban | hospital | inpatient | 341 | axillary (37.5), 48h |
| [37] | Crump**^¶^** | multiple | Tanzania | Eastern | 2007 | 2008 | cross-sectional | urban | hospital | inpatient | 244 | oral (38) |
| [38] | Crump | multiple | Egypt | Northern | 2001 | 2001 | surveillance | rural | hospital/community | community/hospital | 449 | NA |
| [39] | D´Acremont | multiple | Tanzania | Eastern | 2008 | 2008 | NA | urban/rural | hospital | outpatient | 1,005 | axillary (38), <7d |
| [40] | Dahmane | multiple | Sierra Leone | Western | 2011 | 2012 | cross-sectional/historical | rural | hospital | inpatient | 84 | no location (38) |
| [41] | de Araújo Lobo | single | Sierra Leone | Western | 1996 | 1980 | cross-sectional/ archived samples | NA | hospital | inpatient | 149 | 7d |
| [42] | de Geus | single | Kenya | Eastern | 1969 | 1969 | cross-sectional | NA | hospital | outpatient | 281 | no location (38) |
| [43] | Decuypere | multiple | Burkina Faso | Western | NA | NA | NA | rural | hospital | inpatient | 61 | axillary (38) |
| [44] | Dougle | multiple | Kenya | Eastern | 1994 | 1994 | cross-sectional | rural | hospital | inpatient | 229 | axillary (38) |
| [45] | El-Amin | multiple | Sudan | Northern | 2011 | 2011 | cross-sectional | urban | hospital | inpatient | 104 | NA |
| [46] | Elhelw | single | Egypt | Northern | 2008 | 2009 | NA | rural | community | NA | 51 | no location (38), 3d |
| [47] | Enyuma | single | Nigeria | Western | 2010 | 2011 | cross-sectional | NA | hospital | inpatient | 150 | no location (37.5) |
| [48] | Ephraim | multiple | Ghana | Western | 2012 | 2013 | cross-sectional | NA | hospital | outpatient | 284 | axillary (38) |
| [49] | Feleke | multiple | Ethiopia | Eastern | 2011 | 2011 | cross-sectional | NA | primary healthcare facility | NA | 280 | no location (37.5), 48h |
| [50] | Ferede | single | Ethiopia | Eastern | 2016 | 2017 | cross-sectional | urban/rural | hospital | inpatient/outpatient | 600 | no location (38) |
| [51] | Furukawa | single | Kenya | Eastern | 2009 | 2012 | cross-sectional | urban | community/primary healthcare facility | inpatient/community | 97 | axillary (38) |
| [52] | Ghiorghis | multiple | Ethiopia | Eastern | 1988 | 1989 | cross-sectional | urban | hospital | outpatient | 634 | axillary (39), 48-72h |
| [53] | Gordon | single | Malawi | Eastern | 1999 | 2004 | cross-sectional | urban | hospital | inpatient | 62,878 | axillary (37.5) |
| [54] | Gordon | multiple | Malawi | Eastern | 1997 | 1998 | cross-sectional | urban | hospital | inpatient | 9,298 | no location (37.5) |
| [55] | Guillebaud | multiple | Madagascar | Eastern | 2014 | 2015 | cross-sectional | NA | primary healthcare facility | outpatient | 682 | axillary (37.5) |
| [56] | Hagen**^\|\|^** | single | Madagascar | Eastern | 2011 | 2013 | cross-sectional | urban/rural | primary healthcare facility | inpatient/outpatient | 1,009 | no location (38.5), 72h |
| [57] | Hassanain | multiple | Sudan | Northern | 2007 | 2007 | cross-sectional | NA | hospital | NA | 149 | no location (37.5) |
| [58] | Hercik | multiple | Tanzania | Eastern | 2014 | 2014 | cross-sectional | rural | hospital/primary healthcare facility | inpatient/outpatient | 191 | axillary (37.5), <5d |
| [59] | Hercik | multiple | Tanzania | Eastern | 2014 | 2015 | cross-sectional | rural | hospital | inpatient/outpatient | 842 | axillary (37.5) |
| [60] | Hertz**^¶^** | multiple | Tanzania | Eastern | 2007 | 2008 | cross-sectional | urban/rural | hospital | inpatient | 700 | axillary (37.5), oral (38), rectal (38), 48h |
| [61] | Hogan | multiple | Ghana | Western | 2013 | 2015 | cross-sectional | rural | hospital | inpatient | 1,238 | tympanic (38) |
| [62] | Hyams | multiple | Sudan | Northern | 1984 | 1984 | cross-sectional | urban | hospital | inpatient | 100 | no location (37.8) |
| [63] | Ibeneme | multiple | Nigeria | Western | 2010 | 2010 | cross-sectional | urban | hospital | outpatient | 200 | NA |
| [64] | Isendahl | multiple | Guinea-Bissau | Western | 2010 | 2010 | cross-sectional | urban | hospital | inpatient/outpatient | 372 | axillary (38) |
| [65] | Kaabia | multiple | Tunisia | Northern | 2004 | 2004 | cross-sectional/paired samples | urban/rural | hospital | inpatient | 47 | NA |
| [66] | Karsas | multiple | South Africa | Southern | 2013 | 2014 | cross-sectional | urban | hospital | inpatient | 51 | axillary (38) |
| [67] | Kassa-Kelembho | multiple | Central African Republic | Central | 1998 | 1998 | cross-sectional | NA | hospital | inpatient | 15 | NA |
| [68] | Kibuuka | multiple | Uganda | Eastern | 2012 | 2012 | cross-sectional | urban | hospital | inpatient | 235 | axillary (37.5), 24h |
| [69] | Kiemde | multiple | Burkina Faso | Western | 2015 | 2015 | cross-sectional | rural | hospital/primary healthcare facility | NA | 143 | axillary (37.5) |
| [70] | Kinimi | multiple | Tanzania | Eastern | 2015 | 2015 | cross-sectional | rural | hospital/primary healthcare facility | outpatient | 400 | axillary (38), rectal (38), 2-5d |
| [71] | Ki-Zerbo | multiple | Burkina Faso | Western | 1995 | 1995 | cross-sectional/paired samples | urban | hospital | inpatient | 183 | no location (38) |
| [72] | L'Azou | single | Côte d’Ivoire | Western | 2011 | 2012 | cross-sectional | urban | hospital | inpatient/outpatient | 796 | no location (38), 7d |
| [73] | Lepage | multiple | Rwanda | Eastern | 1984 | 1985 | cross-sectional | urban | hospital | inpatient/outpatient | 900 | rectal (39) |
| [74] | Lewis | multiple | Malawi | Eastern | 2000 | 2000 | cross-sectional | urban | hospital | inpatient | 59 | NA |
| [75] | Ley | single | Tanzania | Eastern | 2008 | 2009 | cross-sectional | NA | hospital | inpatient | 1,680 | no location (37.5) |
| [76] | Lundgren | multiple | Tanzania | Eastern | 2006 | 2009 | cross-sectional | urban | hospital | inpatient | 369 | tympanic (37.5) |
| [77] | Mahende | multiple | Tanzania | Eastern | 2013 | 2013 | cross-sectional | NA | hospital | outpatient | 808 | axillary (37.5), 48h |
| [78] | Maina | multiple | Kenya | Eastern | 2011 | 2012 | cross-sectional/ paired samples | NA | hospital | inpatient | 281 | no location (37.5) |
| [79] | Marks**^‡‡^** | multiple | Sudan | Northern | 2012 | 2013 | surveillance | urban | primary healthcare facility | outpatient | 644 | NA |
|  | Marks**^‡‡^** | multiple | Ethiopia | Eastern | 2012 | 2014 | surveillance | semi-urban/rural | hospital/primary healthcare facility | inpatient/outpatient | 847 | NA |
|  | Marks**^‡‡^** | multiple | Kenya | Eastern | 2012 | 2013 | surveillance | urban | primary healthcare facility | outpatient | 1,251 | NA |
|  | Marks**^‡‡^** | multiple | Tanzania | Eastern | 2011 | 2013 | surveillance | urban/rural | hospital | inpatient/outpatient | 680 | NA |
|  | Marks**^‡‡^** | multiple | Madagascar | Eastern | 2011 | 2013 | surveillance | urban/rural | primary healthcare facility | outpatient | 2,477 | NA |
|  | Marks**^‡‡^** | multiple | South Africa | Southern | 2012 | 2014 | surveillance | urban | hospital | inpatient | 1,128 | NA |
|  | Marks**^‡‡^** | multiple | Ghana | Western | 2010 | 2012 | surveillance | urban/rural | hospital | inpatient | 2,651 | NA |
|  | Marks**^‡‡^** | multiple | Burkina Faso | Western | 2012 | 2013 | surveillance | semi-urban | hospital/primary healthcare facility | inpatient/outpatient | 1,674 | NA |
|  | Marks**^‡‡^** | multiple | Guinea-Bissau | Western | 2011 | 2013 | surveillance | urban | hospital/primary healthcare facility | inpatient/outpatient | 1,021 | NA |
|  | Marks**^‡‡^** | multiple | Senegal | Western | 2011 | 2013 | surveillance | urban | hospital/primary healthcare facility | inpatient/outpatient | 1,058 | NA |
| [80] | Masakhwe | single | Kenya | Eastern | NA | NA | cross-sectional | urban/rural | hospital | outpatient | 796 | no location (38) |
| [81] | Massangaiae | single | Mozambique | Eastern | 2014 | 2014 | cross-sectional/outbreak investigation | urban | hospital/community | outpatient | 193 | NA |
| [82] | Mava | multiple | Nigeria | Western | 2005 | 2008 | cross-sectional | urban | hospital | inpatient/outpatient | 250 | axillary (37.5) |
| [83] | Maze^**^ | single | Tanzania | Eastern | 2012 | 2014 | cross-sectional/paired | urban/rural/peri-urban | hospital | inpatient/outpatient | 1,273 | axillary (37.5), oral (38), rectal (38), tympanic (38), 72h |
| [84] | McCarthy | multiple | Sudan | Northern | 1988 | 1988 | cross-sectional/paired samples | urban | hospital | outpatient | 196 | no location (37.8) |
| [85] | McDonald | single | Malawi | Eastern | 1997 | 1997 | cross-sectional | urban | hospital | inpatient | 27 | NA |
| [86] | Meremo | single | Tanzania | Eastern | 2011 | 2011 | cross-sectional | urban | hospital | inpatient | 190 | NA |
| [87] | Moore | multiple | Nigeria | Western | 1964 | 1970 | surveillance | urban/rural | community | outpatient | 12,613 | NA |
| [88] | Mourembou | single | Gabon | Central | 2013 | 2014 | cross-sectional | urban/rural | hospital/research unit | outpatient | 410 | axillary (37.5) |
| [89] | Msaki | multiple | Tanzania | Eastern | 2011 | 2011 | cross-sectional | urban | primary healthcare facility | outpatient | 231 | axillary (37.5) |
| [90] | Mtove | multiple | Tanzania | Eastern | 2006 | 2010 | cross-sectional | rural | hospital | inpatient | 6,836 | axillary (37.5) |
| [91] | Muianga | single | Mozambique | Eastern | 2015 | 2016 | cross-sectional/paired samples | urban | primary healthcare facility | outpatient | 300 | axillary (37.6), 5d |
| [92] | Muloki | single | Uganda | Eastern | 2014 | 2015 | cross-sectional | NA | hospital | outpatient | 251 | NA |
| [93] | Musa-Aisien | multiple | Nigeria | Western | 1999 | 1999 | cross-sectional | urban | hospital | inpatient/outpatient | 300 | no location (38) |
| [94] | Musicha | multiple | Malawi | Eastern | 1998 | 2016 | surveillance | urban | hospital | inpatient | 194,539 | axillary (37.5) |
| [95] | Mustafa | single | Sudan | Northern | NA | NA | cross-sectional | NA | hospital | NA | 1,000 | 2w |
| [96] | Nadjm | multiple | Tanzania | Eastern | 2007 | 2007 | surveillance | rural | hospital | inpatient | 111 | axillary (37.5) |
| [97] | Nas | multiple | Nigeria | Western | 2015 | 2015 | cross-sectional | urban/rural | primary healthcare facility | outpatient | 125 | NA |
| [98] | Nashed | single | Sudan | Northern | 1988 | 1988 | cross-sectional | NA | hospital | inpatient | 196 | NA |
| [99] | Ndip**^§§^** | single | Cameroon | Central | 2003 | 2003 | cross-sectional | NA | primary healthcare facility | outpatient | 118 | NA |
| [100] | Ndip**^§§^** | single | Cameroon | Central | 2003 | 2003 | cross-sectional | NA | primary healthcare facility | outpatient | 118 | NA |
| [101] | Ngoi^¶¶^ | multiple | Kenya | Eastern | 2014 | 2015 | cross-sectional | peri-urban | primary healthcare facility | outpatient | 51 | axillary (37.5) |
| [102] | Ngoi^¶¶^ | multiple | Kenya | Eastern | 2014 | 2015 | cross-sectional | peri-urban | primary healthcare facility | outpatient | 489 | axillary (37.5) |
| [103] | Njeru**^\|\|\|\|^** | single | Kenya | Eastern | 2014 | 2015 | cross-sectional | NA | hospital | outpatient | 1,067 | no location (38) |
| [104] | Njeru**^\|\|\|\|^** | single | Kenya | Eastern | 2014 | 2015 | cross-sectional | NA | hospital | outpatient | 1,067 | no location (38) |
| [105] | Ocheke | multiple | Nigeria | Western | 2012 | 2012 | cross-sectional | NA | hospital | outpatient | 303 | axillary (37.5), <2w |
| [106] | Okwara | multiple | Kenya | Eastern | 2001 | 2001 | cross-sectional | urban | hospital | outpatient | 264 | no location (37) |
| [107] | O'Meara | multiple | Kenya | Eastern | 2011 | 2012 | cross-sectional | peri-urban | hospital | outpatient | 354 | no location (37.5), same day |
| [108] | Onoja | multiple | Nigeria | Western | 2014 | 2014 | cross-sectional | urban | hospital | outpatient | 274 | no location (38) |
| [109] | Onubogu | multiple | Nigeria | Western | 2010 | 2011 | cross-sectional | urban | hospital | outpatient | 362 | axillary (37.5) |
| [110] | Onyango | multiple | Kenya | Eastern | 2004 | 2005 | cross-sectional | rural | hospital | NA | 59 | no location (38) |
| [111] | O'Shea | multiple | Sierra Leone | Western | 2014 | 2015 | cross-sectional/ retrospective | NA | treatment unit | inpatient | 91 | no location (38) |
| [112] | Park**^‡‡^** | multiple | Madagascar | Eastern | 2010 | 2014 | surveillance | urban/rural | primary healthcare facility | outpatient | 2,477 | NA |
|  | Park**^‡‡^** | multiple | Senegal | Western | 2010 | 2014 | surveillance | urban | hospital/primary healthcare facility | inpatient/outpatient | 1,058 | NA |
|  | Park**^‡‡^** | multiple | South Africa | Southern | 2010 | 2014 | surveillance | urban | hospital | inpatient | 1,128 | NA |
|  | Park**^‡‡^** | multiple | Sudan | Northern | 2010 | 2014 | surveillance | urban | primary healthcare facility | outpatient | 644 | NA |
|  | Park**^‡‡^** | multiple | Tanzania | Eastern | 2010 | 2014 | surveillance | urban/rural | hospital | inpatient/outpatient | 680 | NA |
| [113] | Peters | multiple | Malawi | Eastern | 2000 | 2000 | cross-sectional | urban | hospital | inpatient | 61 | axillary (37.4), 4d |
| [114] | Petit | multiple | Kenya | Eastern | 1987 | 1990 | cross-sectional | rural | hospital | inpatient | 449 | no location (38) |
|  | Petit | multiple | Ghana | Western | 1987 | 1990 | cross-sectional | rural | hospital | inpatient | 190 | NA |
| [115] | Prabhu**^¶^** | multiple | Tanzania | Eastern | 2007 | 2008 | cross-sectional | urban | hospital | inpatient | 483 | axillary (37.5), oral (38), rectal (37.5), 48h |
| [116] | Preziosi | multiple | Mozambique | Eastern | 2011 | 2014 | cross-sectional | urban | hospital | inpatient | 841 | no location (38) axillary (38), 24h |
| [117] | Rabasa | multiple | Nigeria | Western | 2004 | 2005 | cross-sectional | NA | hospital | NA | 145 | axillary (37.5) |
| [118] | Ribeiro | single | Mozambique | Eastern | 2012 | 2015 | cross-sectional/paired samples | urban/suburban | hospital | outpatient | 373 | NA |
| [119] | Ridde | single | Burkina Faso | Western | 2013 | 2014 | cross-sectional | urban | primary healthcare facility | NA | 379 | axillary (38), <1w |
| [120] | Schoepp | multiple | Sierra Leone | Western | 2006 | 2008 | cross-sectional/outbreak investigation | NA | hospital | inpatient | 253 | no location (38) |
| [121] | Sigauque | multiple | Mozambique | Eastern | 2001 | 2006 | cross-sectional | rural | hospital | inpatient | 19,896 | axillary (37.5), 24h |
| [122] | Socolovschi | single | Senegal | Western | 2008 | 2009 | cross-sectional | rural | community | community | 134 | no location (37.5) |
| [123] | Sow | single | Mali | Western | 2002 | 2004 | cross-sectional | urban | hospital | inpatient | 3,592 | axillary (39) |
| [124] | Ssali | multiple | Uganda | Eastern | 1997 | 1997 | cross-sectional | urban | hospital | inpatient | 71 | axillary (38) |
| [125] | Thiga | multiple | Kenya | Eastern | 1997 | 1998 | cross-sectional | NA | hospital | NA | 2,225 | no location (38) |
| [126] | Thompson | multiple | Kenya | Eastern | 2009 | 2011 | cross-sectional/retrospective | rural | hospital | outpatient | 1,917 | axillary (38) |
| [127] | Thriemer**^***^** | single | Zanzibar (Tanzania) | Eastern | 2009 | 2010 | cross-sectional | NA | hospital | inpatient/outpatient | 2,209 | tympanic (37.5) |
| [128] | Thriemer**^***^** | multiple | Zanzibar  (Tanzania) | Eastern | 2009 | 2010 | surveillance | rural | hospital | inpatient/outpatient | 2,209 | tympanic (37.5) |
| [129] | Waggoner | multiple | Kenya | Eastern | 2014 | 2015 | cross-sectional | urban/rural | hospital/primary healthcare facility | outpatient | 385 | no location (38), <5d |
| [130] | Walsh | multiple | Malawi | Eastern | 1996 | 1997 | cross-sectional | urban | hospital | inpatient | 365 | axillary (38) |
| [131] | Woodruff | multiple | Sudan | Northern | 1986 | 1986 | cross-sectional/paired samples | urban/rural | hospital | inpatient | 130 | NA |
| [132] | Yimer | single | Ethiopia | Eastern | 2003 | 2003 | cross-sectional | NA | hospital | NA | 59 | NA |
| [133] | Zenebe | multiple | Ethiopia | Eastern | 2009 | 2010 | cross-sectional | urban/rural | hospital | outpatient | 260 | axillary (38) |

NA = not available/applicable

*Only countries included in our analyses are presented

† Non-HIV populations only (when stated in publication)

**^‡^** This included the measurement location(s), body temperature in degrees Celsius (°C), and duration of fever in hours (h), days (d), or weeks (w)

**^§ ¶ || ** †† ‡‡ §§ ¶¶ |||| ***^** Studies were based on the same population

# References of included studies

1. Aarsland SJ, Castellanos-Gonzalez A, Lockamy KP, Mulu-Droppers R, Mulu M, White AC, et al. Treatable bacterial infections are underrecognized causes of fever in Ethiopian children. Am J Trop Med Hyg. 2012;87(1):128-33. doi: 10.4269/ajtmh.2012.12-0171. PubMed PMID: 22764303.

2. Achonduh-Atijegbe OA, Mfuh KO, Mbange AH, Chedjou JP, Taylor DW, Nerurkar VR, et al. Prevalence of malaria, typhoid, toxoplasmosis and rubella among febrile children in Cameroon. BMC Infect Dis. 2016;16(1):658.

3. Adam A, Seidahmed OM, Weber C, Schnierle B, Schmidt-Chanasit J, Reiche S, et al. Low seroprevalence indicates vulnerability of eastern and central Sudan to infection with chikungunya virus. Vector Borne Zoonotic Dis. 2016;16(4):290-1.

4. Adedayo F, Nioma I, Olanrewaju MB, Adeyinka A, Ebele A. Serological evidence of recent dengue virus infection among febrile children in a semi arid zone. Am J Infect Dis. 2013;9(1):7.

5. Adjei O, Opoku C. Urinary tract infections in African infants. Int J Antimicrob Agents. 2004;24:32-4.

6. Afifi S, Earhart K, Azab MA, Youssef FG, El Sakka H, Wasfy M, et al. Hospital-based surveillance for acute febrile illness in Egypt: a focus on community-acquired bloodstream infections. Am J Trop Med Hyg. 2005;73(2):392-9. Epub 2005/08/17. PubMed PMID: 16103611.

7. Akhuemokhan OC, Ewah-Odiase RO, Akpede N, Ehimuan J, Adomeh DI, Odia I, et al. Prevalence of Lassa Virus Disease (LVD) in Nigerian children with fever or fever and convulsions in an endemic area. PLoS Negl Trop Dis. 2017;11(7):e0005711.

8. Akpede GO, Abiodun PO, Sykes RM. Acute fevers of unknown origin in young children in the tropics. J Pediatr. 1993;122(1):79-81. Epub 1993/01/01. doi: 10.1016/s0022-3476(05)83491-2. PubMed PMID: 8419618.

9. Akpede GO, Abiodun PO, Sykes RM. Pattern of infections in children under-six years old presenting with convulsions associated with fever of acute onset in a children's emergency room in Benin City, Nigeria. J Trop Pediatr. 1993;39(1):11-5. Epub 1993/02/01. doi: 10.1093/tropej/39.1.11. PubMed PMID: 8445682.

10. Akpede GO, Abiodun PO, Sykes RM. Relative contribution of bacteraemia and malaria to acute fever without localizing signs of infection in under-five children. J Trop Pediatr. 1992;38(6):295-8. Epub 1991/12/01. doi: 10.1093/tropej/38.6.295. PubMed PMID: 1844088.

11. Animut A, Mekonnen Y, Shimelis D, Ephraim E. Febrile illnesses of different etiology among outpatients in four health centers in Northwestern Ethiopia. Jpn J Infect Dis. 2009;62(2):107-10. Epub 2009/03/24. PubMed PMID: 19305049.

12. António VS, Muianga AF, Wieseler J, Pereira SA, Monteiro VO, Mula F, et al. Seroepidemiology of chikungunya virus among febrile patients in eight health facilities in central and northern Mozambique, 2015–2016. Vector Borne Zoonotic Dis. 2018;18(6):311-6.

13. Archibald LK, den Dulk MO, Pallangyo KJ, Reller LB. Fatal *Mycobacterium tuberculosis* bloodstream infections in febrile hospitalized adults in Dar es Salaam, Tanzania. Clin Infect Dis. 1998;26(2):290-6. Epub 1998/03/21. doi: 10.1086/516297. PubMed PMID: 9502444.

14. Archibald LK, McDonald LC, Nwanyanwu O, Kazembe P, Dobbie H, Tokars J, et al. A hospital-based prevalence survey of bloodstream infections in febrile patients in Malawi: implications for diagnosis and therapy. J Infect Dis. 2000;181(4):1414-20. Epub 2000/04/14. doi: 10.1086/315367. PubMed PMID: 10762572.

15. Ayoola O, Adeyemo A, Osinusi K. Aetiological agents, clinical features and outcome of septicaemia in infants in Ibadan. West Afr J Med. 2003;22(1):30-4.

16. Ba O, Fleming JA, Dieye Y, wa Mutombo BM, Ba M, Cisse MF, et al. Hospital surveillance of childhood bacterial meningitis in Senegal and the introduction of *Haemophilus influenzae* type b conjugate vaccine. Am J Trop Med Hyg. 2010;83(6):1330-5.

17. Baba M, Logue CH, Oderinde B, Abdulmaleek H, Williams J, Lewis J, et al. Evidence of arbovirus co-infection in suspected febrile malaria and typhoid patients in Nigeria. J Infect Dev Ctries. 2013;7(01):051-9.

18. Baudin M, Jumaa AM, Jomma HJ, Karsany MS, Bucht G, Näslund J, et al. Association of Rift Valley fever virus infection with miscarriage in Sudanese women: a cross-sectional study. Lancet Global Health. 2016;4(11):e864-e71.

19. Bell M, Archibald LK, Nwanyanwu O, Dobbie H, Tokars J, Kazembe PN, et al. Seasonal variation in the etiology of bloodstream infections in a febrile inpatient population in a developing country. Int J Infect Dis. 2001;5(2):63-9. Epub 2001/07/27. doi: 10.1016/s1201-9712(01)90027-x. PubMed PMID: 11468099.

20. Bevilacqua N, Pane S, Vairo F, Nicastri E, Paglia MG, Ame SM, et al. Accuracy of indirect haemagglutination and western blot assays for the detection of anti-*Schistosoma* antibodies in non-severe febrile patients in two Tanzanian hospitals. Scand J Infect Dis. 2012;44(6):453-8.

21. Biggs HM, Bui DM, Galloway RL, Stoddard RA, Shadomy SV, Morrissey AB, et al. Leptospirosis among hospitalized febrile patients in northern Tanzania. Am J Trop Med Hyg. 2011;85(2):275-81. Epub 2011/08/05. doi: 10.4269/ajtmh.2011.11-0176. PubMed PMID: 21813847; PubMed Central PMCID: PMCPMC3144825 Hygiene annual meeting, Atlanta, GA, 3–7 November 2010, abstract 856.

22. Biscornet L, Dellagi K, Pagès F, Bibi J, de Comarmond J, Mélade J, et al. Human leptospirosis in Seychelles: A prospective study confirms the heavy burden of the disease but suggests that rats are not the main reservoir. PLoS Negl Trop Dis. 2017;11(8):e0005831. Epub 2017/08/29. doi: 10.1371/journal.pntd.0005831. PubMed PMID: 28846678; PubMed Central PMCID: PMCPMC5591009.

23. Boone I, Henning K, Hilbert A, Neubauer H, Von Kalckreuth V, Dekker DM, et al. Are brucellosis, Q fever and melioidosis potential causes of febrile illness in Madagascar? Acta Trop. 2017;172:255-62.

24. Botros BAM, Soliman AK, Salib AW, Olson J, Hibbs RG, Williams JC, et al. *Coxiella burnetii* antibody prevalences among human populations in north-east Africa determined by enzyme immunoassay. J Trop Med Hyg. 1995;98(3):173-8.

25. Bouley AJ, Biggs HM, Stoddard RA, Morrissey AB, Bartlett JA, Afwamba IA, et al. Brucellosis among hospitalized febrile patients in northern Tanzania. Am J Trop Med Hyg. 2012;87(6):1105-11. Epub 2012/10/24. doi: 10.4269/ajtmh.2012.12-0327. PubMed PMID: 23091197; PubMed Central PMCID: PMCPMC3516083.

26. Bouyou-Akotet MK, Mawili-Mboumba DP, Kendjo E, Ekouma AE, Raouf OA, Allogho EE, et al. Complicated malaria and other severe febrile illness in a pediatric ward in Libreville, Gabon. BMC Infect Dis. 2012;12(1):216.

27. Brown B, Asinobi A, Fatunde O, Osinusi K, Fasina N. Evaluation of the nitrite test in screening for urinary tract infection in febrile children with sickle cell anaemia. Niger J Paediatr. 2004;31(1):10-3.

28. Brown V, Larouze B, Desve G, Rousset J, Thibon M, Fourrier A, et al. Clinical presentation of louse-borne relapsing fever among Ethiopian refugees in northern Somalia. Ann Trop Med Parasitol. 1988;82(5):499-502.

29. Camara N, Ngasala B, Leyna G, Abade A, Rumisha SF, Oriyo NM, et al. Socio-demographic determinants of dengue infection during an outbreak in Dar es Salaam City, Tanzania. Tanzan J Health Res. 2018;20(2).

30. Cash-Goldwasser S, Maze MJ, Rubach MP, Biggs HM, Stoddard RA, Sharples KJ, et al. Risk factors for human brucellosis in northern Tanzania. Am J Trop Med Hyg. 2018;98(2):598-606.

31. Chau R, Bhatt N, Manhiça I, Cândido S, de Deus N, Guiliche O, et al. First serological evidence of hantavirus among febrile patients in Mozambique. Int J Infect Dis. 2017;61:51-5. doi: 10.1016/j.ijid.2017.06.001.

32. Chipwaza B, Mhamphi GG, Ngatunga SD, Selemani M, Amuri M, Mugasa JP, et al. Prevalence of bacterial febrile illnesses in children in Kilosa district, Tanzania. PLoS Negl Trop Dis. 2015;9(5):e0003750-e. doi: 10.1371/journal.pntd.0003750. PubMed PMID: 25955522.

33. Chipwaza B, Mugasa JP, Selemani M, Amuri M, Mosha F, Ngatunga SD, et al. Dengue and Chikungunya fever among viral diseases in outpatient febrile children in Kilosa district hospital, Tanzania. PLoS Negl Trop Dis. 2014;8(11).

34. Christopher A, Mshana SE, Kidenya BR, Hokororo A, Morona D. Bacteremia and resistant gram-negative pathogens among under-fives in Tanzania. Ital J Pediatr. 2013;39(1):27.

35. Commey J, Quarm-Goka B, Agyepong I. Persistent fever in severe malaria in children. Cent Afr J Med. 1994;40(9):257-60.

36. Crump JA, Ramadhani HO, Morrissey AB, Msuya LJ, Yang LY, Chow SC, et al. Invasive bacterial and fungal infections among hospitalized HIV-infected and HIV-uninfected children and infants in northern Tanzania. Trop Med Int Health. 2011;16(7):830-7. Epub 2011/04/08. doi: 10.1111/j.1365-3156.2011.02774.x. PubMed PMID: 21470347; PubMed Central PMCID: PMCPMC3227789.

37. Crump JA, Ramadhani HO, Morrissey AB, Saganda W, Mwako MS, Yang LY, et al. Invasive bacterial and fungal infections among hospitalized HIV-infected and HIV-uninfected adults and adolescents in northern Tanzania. Clin Infect Dis. 2011;52(3):341-8. Epub 2011/01/11. doi: 10.1093/cid/ciq103. PubMed PMID: 21217181; PubMed Central PMCID: PMCPMC3106248.

38. Crump JA, Youssef FG, Luby SP, Wasfy MO, Rangel JM, Taalat M, et al. Estimating the incidence of typhoid fever and other febrile illnesses in developing countries. Emerg Infect Dis. 2003;9(5):539-44. doi: 10.3201/eid0905.020428. PubMed PMID: 12737736.

39. D'Acremont V, Kilowoko M, Kyungu E, Philipina S, Sangu W, Kahama-Maro J, et al. Beyond malaria—causes of fever in outpatient Tanzanian children. N Engl J Med. 2014;370(9):809-17.

40. Dahmane A, Van Griensven J, Van Herp M, Van den Bergh R, Nzomukunda Y, Prior J, et al. Constraints in the diagnosis and treatment of Lassa fever and the effect on mortality in hospitalized children and women with obstetric conditions in a rural district hospital in Sierra Leone. Trans R Soc Trop Med Hyg. 2014;108(3):126-32.

41. de Araujo Lobo JM, Mores CN, Bausch DG, Christofferson RC. Serological evidence of under-reported dengue circulation in Sierra Leone. PLoS Negl Trop Dis. 2016;10(4).

42. de Geus A, Wolff JW, Timmer VEA. Clinical leptospirosis in Kenya, II. A field study in Nyanza Province. East Afr Med J. 1977;54(3):125-32.

43. Decuypere S, Maltha J, Deborggraeve S, Rattray NJW, Issa G, Bérenger K, et al. Towards improving point-of-care diagnosis of non-malaria febrile illness: A metabolomics approach. PLoS Negl Trop Dis. 2016;10(3):e0004480. doi: 10.1371/journal.pntd.0004480.

44. Dougle ML, Hendriks ER, Sanders EJ, Dorigo-Zetsma JW. Laboratory investigations in the diagnosis of septicaemia and malaria. East Afr Med J. 1997;74(6):353-6. Epub 1997/06/01. PubMed PMID: 9487395.

45. El-Amin EO, Elbashir MI, Elamin OE, Mukhtar Y, Abdo H, Abdul-Rahman I, et al. The underlying aetiologies of coma in febrile Sudanese children. Trans R Soc Trop Med Hyg. 2013;107(5):307-12. Epub 2013/04/16. doi: 10.1093/trstmh/trt013. PubMed PMID: 23584374.

46. Elhelw RA, El-Enbaawy MI, Samir A. Lyme borreliosis: A neglected zoonosis in Egypt. Acta Trop. 2014;140:188-92. Epub 2014/09/23. doi: 10.1016/j.actatropica.2014.09.005. PubMed PMID: 25239124.

47. Enyuma C, Meremikwu M, Udo J, Anah M, Asindi A. Malaria parasite positivity among febrile neonates. Niger J Paediatr. 2014;41(4):321-5.

48. Ephraim R, Nyame M, Sakyi S, Antoh E, Simpong D. Co-existence of malaria and urinary tract infection among children under five: A cross-sectional study of the Assin-South Municipality, Ghana. J Medical Biomed Sci. 2013;2(4):35-41.

49. Feleke SM, Animut A, Belay M. Prevalence of malaria among acute febrile patients clinically suspected of having malaria in the Zeway Health Center, Ethiopia. Jpn J Infect Dis. 2015;68(1):55-9. Epub 2014/11/26. doi: 10.7883/yoken.JJID.2013.062. PubMed PMID: 25420658.

50. Ferede G, Tiruneh M, Abate E, Wondimeneh Y, Damtie D, Gadisa E, et al. A serologic study of dengue in northwest Ethiopia: Suggesting preventive and control measures. PLoS Negl Trop Dis. 2018;12(5).

51. Furukawa NW, Teshale EH, Cosmas L, Ochieng M, Gikunju S, Fields BS, et al. Serologic evidence for hepatitis E virus infection among patients with undifferentiated acute febrile illness in Kibera, Kenya. J Clin Virol. 2016;77:106-8. Epub 2016/03/02. doi: 10.1016/j.jcv.2016.02.021. PubMed PMID: 26925954; PubMed Central PMCID: PMCPMC4861756.

52. Ghiorghis B, Geyid A, Haile M. Bacteraemia in febrile out-patient children. East Afr Med J. 1992;69(2):74-7.

53. Gordon MA, Graham SM, Walsh AL, Wilson L, Phiri A, Molyneux E, et al. Epidemics of invasive *Salmonella enterica* serovar Enteritidis and *S. enterica* serovar Typhimurium infection associated with multidrug resistance among adults and children in Malawi. Clin Infect Dis. 2008;46(7):963-9. Epub 2008/05/01. doi: 10.1086/529146. PubMed PMID: 18444810.

54. Gordon MA, Walsh AL, Chaponda M, Soko D, Mbvwinji M, Molyneux ME, et al. Bacteraemia and mortality among adult medical admissions in Malawi – predominance of non-typhi salmonellae and *Streptococcus pneumoniae*. J Infect. 2001;42(1):44-9. Epub 2001/03/13. doi: 10.1053/jinf.2000.0779. PubMed PMID: 11243753.

55. Guillebaud J, Bernardson B, Randriambolamanantsoa TH, Randrianasolo L, Randriamampionona JL, Marino CA, et al. Study on causes of fever in primary healthcare center uncovers pathogens of public health concern in Madagascar. PLoS Negl Trop Dis. 2018;12(7):e0006642.

56. Hagen RM, Frickmann H, Ehlers J, Krüger A, Margos G, Hizo-Teufel C, et al. Presence of *Borrelia* spp. DNA in ticks, but absence of *Borrelia* spp. and of *Leptospira* spp. DNA in blood of fever patients in Madagascar. Acta Trop. 2018;177:127-34. Epub 2017/10/08. doi: 10.1016/j.actatropica.2017.10.002. PubMed PMID: 28986249.

57. Hassanain AM, Noureldien W, Karsany MS, Saeed ES, Aradaib IE, Adam I. Rift Valley fever among febrile patients at New Halfa hospital, eastern Sudan. Virol J. 2010;7(1):97.

58. Hercik C, Cosmas L, Mogeni OD, Wamola N, Kohi W, Houpt E, et al. A combined syndromic approach to examine viral, bacterial, and parasitic agents among febrile patients: A pilot study in Kilombero, Tanzania. Am J Trop Med Hyg. 2018;98(2):625-32. Epub 2017/12/28. doi: 10.4269/ajtmh.17-0421. PubMed PMID: 29280432; PubMed Central PMCID: PMCPMC5929188.

59. Hercik C, Cosmas L, Mogeni OD, Wamola N, Kohi W, Omballa V, et al. A diagnostic and epidemiologic investigation of acute febrile illness (AFI) in Kilombero, Tanzania. PLoS One. 2017;12(12):e0189712. doi: 10.1371/journal.pone.0189712.

60. Hertz JT, Munishi OM, Ooi EE, Howe S, Lim WY, Chow A, et al. Chikungunya and dengue fever among hospitalized febrile patients in northern Tanzania. Am J Trop Med Hyg. 2012;86(1):171-7.

61. Hogan B, Eibach D, Krumkamp R, Sarpong N, Dekker D, Kreuels B, et al. Malaria coinfections in febrile pediatric inpatients: a hospital-based study from Ghana. Clin Infect Dis. 2018;66(12):1838-45.

62. Hyams KC, Oldfield EC, Scott RM, Bourgeois AL, Gardiner H, Pazzaglia G, et al. Evaluation of febrile patients in Port Sudan, Sudan: isolation of dengue virus. Am J Trop Med Hyg. 1986;35(4):860-5. Epub 1986/07/01. doi: 10.4269/ajtmh.1986.35.860. PubMed PMID: 3728800.

63. Ibeneme C, Oguonu T, Ikefuna A, Okafor H, Ozumba U. Bacteriology of urinary tract infection and antimicrobial sensitivities in under-five children in Enugu. Niger J Paediatr. 2014;41(3):188-93.

64. Isendahl J, Manjuba C, Rodrigues A, Xu W, Henriques-Normark B, Giske CG, et al. Prevalence of community-acquired bacteraemia in Guinea-Bissau: an observational study. BMC Infect Dis. 2014;14:3859-. doi: 10.1186/s12879-014-0715-9. PubMed PMID: 25526763.

65. Kaabia N, Rolain J, Khalifa M, Jazia EB, Bahri F, Raoult D, et al. Serologic study of rickettsioses among acute febrile patients in central Tunisia. Ann N Y Acad Sci. 2006;1078(1):176-9.

66. Karsas M, Becker PJ, Green RJ. Serious bacterial infections in febrile young children: Lack of value of biomarkers. S Afr J Child Health. 2016;10(1):33-6.

67. Kassa-Kelembho E, Mbolidi CD, Service YB, Morvan J, Minssart P. Bacteremia in adults admitted to the Department of Medicine of Bangui Community Hospital (Central African Republic). Acta Trop. 2003;89(1):67-72. Epub 2003/11/26. doi: 10.1016/j.actatropica.2003.09.004. PubMed PMID: 14636984.

68. Kibuuka A, Byakika-Kibwika P, Achan J, Yeka A, Nalyazi JN, Mpimbaza A, et al. Bacteremia among febrile Ugandan children treated with antimalarials despite a negative malaria test. Am J Trop Med Hyg. 2015;93(2):276-80. Epub 2015/06/10. doi: 10.4269/ajtmh.14-0494. PubMed PMID: 26055736; PubMed Central PMCID: PMCPMC4530747.

69. Kiemde F, Tahita MC, Lompo P, Rouamba T, Some AM, Tinto H, et al. Treatable causes of fever among children under five years in a seasonal malaria transmission area in Burkina Faso. Infect Dis Poverty. 2018;7(1):60.

70. Kinimi E, Patrick BN, Misinzo G. Serological evidence of chikungunya and malaria co-infection among febrile patients seeking health care in Karagwe district, Tanzania. Tanzan J Health Res. 2018;20(4).

71. Ki-Zerbo G, Tall F, Nagalo K, Ledru E, Durand G, Patey O. Séroprévalence des rickettsioses et de la fièvre Q chez les patients fébriles à l'hôpital de Bobo-Dioulasso (Burkina Faso). Med Mal Infect. 2000;30(5):270-4.

72. L'Azou M, Succo T, Kamagaté M, Ouattara A, Gilbernair E, Adjogoua E, et al. Dengue: etiology of acute febrile illness in Abidjan, Côte d'Ivoire, in 2011–2012. Trans R Soc Trop Med Hyg. 2015;109(11):717-22.

73. Lepage P, Bogaerts J, Van Goethem C, Ntahorutaba M, Nsengumuremyi F, Hitimana DG, et al. Community-acquired bacteraemia in African children. Lancet. 1987;1(8548):1458-61. Epub 1987/06/27. doi: 10.1016/s0140-6736(87)92207-0. PubMed PMID: 2885453.

74. Lewis DK, Peters RP, Schijffelen MJ, Joaki GR, Walsh AL, Kublin JG, et al. Clinical indicators of mycobacteraemia in adults admitted to hospital in Blantyre, Malawi. Int J Tuberc Lung Dis. 2002;6(12):1067-74. Epub 2003/01/28. PubMed PMID: 12546114.

75. Ley B, Mtove G, Thriemer K, Amos B, von Seidlein L, Hendriksen I, et al. Evaluation of the Widal tube agglutination test for the diagnosis of typhoid fever among children admitted to a rural hospital in Tanzania and a comparison with previous studies. BMC Infect Dis. 2010;10:180. Epub 2010/06/23. doi: 10.1186/1471-2334-10-180. PubMed PMID: 20565990; PubMed Central PMCID: PMCPMC2898821.

76. Lundgren IS, Heltshe SL, Smith AL, Chibwana J, Fried MW, Duffy PE. Bacteremia and malaria in Tanzanian children hospitalized for acute febrile illness. J Trop Pediatr. 2015;61(2):81-5. Epub 2014/12/09. doi: 10.1093/tropej/fmu069. PubMed PMID: 25505140.

77. Mahende C, Ngasala B, Lusingu J, Butichi A, Lushino P, Lemnge M, et al. Bloodstream bacterial infection among outpatient children with acute febrile illness in north-eastern Tanzania. BMC Res Notes. 2015;8:289-. doi: 10.1186/s13104-015-1178-9. PubMed PMID: 26138060.

78. Maina AN, Farris CM, Odhiambo A, Jiang J, Laktabai J, Armstrong J, et al. Q fever, scrub typhus, and rickettsial diseases in children, Kenya, 2011–2012. Emerg Infect Dis. 2016;22(5):883.

79. Marks F, von Kalckreuth V, Aaby P, Adu-Sarkodie Y, El Tayeb MA, Ali M, et al. Incidence of invasive salmonella disease in sub-Saharan Africa: a multicentre population-based surveillance study. Lancet Global Health. 2017;5(3):e310-e23. Epub 2017/02/15. doi: 10.1016/s2214-109x(17)30022-0. PubMed PMID: 28193398; PubMed Central PMCID: PMCPMC5316558.

80. Masakhwe C, Ochanda H, Nyakoe N, Ochiel D, Waitumbi J. Frequency of Epstein-Barr Virus in patients presenting with acute febrile illness in Kenya. PLoS One. 2016;11(5).

81. Massangaie M, Pinto G, Padama F, Chambe G, Da Silva M, Mate I, et al. Clinical and epidemiological characterization of the first recognized outbreak of Dengue virus-type 2 in Mozambique, 2014. Am J Trop Med Hyg. 2016;94(2):413-6. Epub 2015/12/09. doi: 10.4269/ajtmh.15-0543. PubMed PMID: 26643534; PubMed Central PMCID: PMCPMC4751938.

82. Mava Y, Ambe J, Bello M, Watila I, Nottidge V. Urinary tract infection in febrile children with sickle cell anaemia. West Afr J Med. 2011;30(4):268-72.

83. Maze MJ, Cash-Goldwasser S, Rubach MP, Biggs HM, Galloway RL, Sharples KJ, et al. Risk factors for human acute leptospirosis in northern Tanzania. PLoS Negl Trop Dis. 2018;12(6):e0006372-e. doi: 10.1371/journal.pntd.0006372. PubMed PMID: 29879114.

84. McCarthy M, Haberberger R, Salib A, Soliman B, El‐Tigani A, Khalid I, et al. Evaluation of arthropod‐borne viruses and other infectious disease pathogens as the causes of febrile illnesses in the Khartoum Province of Sudan. J Med Virol. 1996;48(2):141-6.

85. McDonald LC, Archibald LK, Rheanpumikankit S, Tansuphaswadikul S, Eampokalap B, Nwanyanawu O, et al. Unrecognised *Mycobacterium tuberculosis* bacteraemia among hospital inpatients in less developed countries. Lancet. 1999;354(9185):1159-63. Epub 1999/10/08. doi: 10.1016/s0140-6736(98)12325-5. PubMed PMID: 10513709.

86. Meremo AJ, Kidenya BR, Mshana SE, Kabangila R, Kataraihya JB. High prevalence of tuberculosis among adults with fever admitted at a tertiary hospital in north-western Tanzania. Tanzan J Health Res. 2012;14(3):183-8. Epub 2012/07/01. doi: 10.4314/thrb.v14i3.4. PubMed PMID: 26591755.

87. Moore DL, Causey OR, Carey DE, Reddy S, Cooke AR, Akinkugbe FM, et al. Arthropod-borne viral infections of man in Nigeria, 1964-1970. Ann Trop Med Parasitol. 1975;69(1):49-64. Epub 1975/03/01. doi: 10.1080/00034983.1975.11686983. PubMed PMID: 1124969.

88. Mourembou G, Lekana-Douki JB, Mediannikov O, Nzondo SM, Kouna LC, Essone JCBB, et al. Possible role of *Rickettsia felis* in acute febrile illness among children in Gabon. Emerg Infect Dis. 2015;21(10):1808.

89. Msaki BP, Mshana SE, Hokororo A, Mazigo HD, Morona D. Prevalence and predictors of urinary tract infection and severe malaria among febrile children attending Makongoro health centre in Mwanza city, North-Western Tanzania. Arch Public Health. 2012;70(1):4.

90. Mtove G, Amos B, Nadjm B, Hendriksen ICE, Dondorp AM, Mwambuli A, et al. Decreasing incidence of severe malaria and community-acquired bacteraemia among hospitalized children in Muheza, north-eastern Tanzania, 2006-2010. Malar J. 2011;10(1):320. doi: 10.1186/1475-2875-10-320.

91. Muianga AF, Watson R, Varghese A, Chongo IS, Ali S, Monteiro V, et al. First serological evidence of Crimean-Congo haemorrhagic fever in febrile patients in Mozambique. Int J Infect Dis. 2017;62:119-23. doi: <https://doi.org/10.1016/j.ijid.2017.07.024>.

92. Muloki HN, Erume J, Owiny DO, Kungu JM, Nakavuma J, Ogeng D, et al. Prevalence and risk factors for brucellosis in prolonged fever patients in post-conflict Northern Uganda. Afr Health Sci. 2018;18(1):22-8.

93. Musa-Aisien A, Ibadin O, Ukoh G, Akpede G. Prevalence and antimicrobial sensitivity pattern in urinary tract infection in febrile under-5s at a children's emergency unit in Nigeria. Ann Trop Paediatr. 2003;23(1):39-45.

94. Musicha P, Cornick JE, Bar-Zeev N, French N, Masesa C, Denis B, et al. Trends in antimicrobial resistance in bloodstream infection isolates at a large urban hospital in Malawi (1998-2016): a surveillance study. Lancet Infect Dis. 2017;17(10):1042-52. Epub 2017/08/19. doi: 10.1016/s1473-3099(17)30394-8. PubMed PMID: 28818544; PubMed Central PMCID: PMCPMC5610140.

95. Mustafa AAA, Hassan HS. Human brucellosis in Khartoum State: A commonly underdiagnosed disease. Sudan J Med Sci. 2010;5(3):213-6.

96. Nadjm B, Mtove G, Amos B, Walker NF, Diefendal H, Reyburn H, et al. Severe febrile illness in adult hospital admissions in Tanzania: a prospective study in an area of high malaria transmission. Trans R Soc Trop Med Hyg. 2012;106(11):688-95. Epub 2012/10/02. doi: 10.1016/j.trstmh.2012.08.006. PubMed PMID: 23022040.

97. Nas F, Ali M, Yahaya A. Malaria and typhoid fever co-infection among febrile patients in Kumbotso Local Government Area Kano, Nigeria. BAJOPAS. 2017;10(2):122-5.

98. Nashed NW, Olson JG, el-Tigani A. Isolation of Batai virus (Bunyaviridae:Bunyavirus) from the blood of suspected malaria patients in Sudan. Am J Trop Med Hyg. 1993;48(5):676-81. Epub 1993/05/01. doi: 10.4269/ajtmh.1993.48.676. PubMed PMID: 8517485.

99. Ndip LM, Fokam EB, Bouyer DH, Ndip RN, Titanji VP, Walker DH, et al. Detection of *Rickettsia africae* in patients and ticks along the coastal region of Cameroon. Am J Trop Med Hyg. 2004;71(3):363-6.

100. Ndip LM, Labruna M, Ndip RN, Walker DH, McBride JW. Molecular and clinical evidence of *Ehrlichia chaffeensis* infection in Cameroonian patients with undifferentiated febrile illness. Ann Trop Med Parasitol. 2009;103(8):719-25. Epub 2009/12/25. doi: 10.1179/000349809x12554106963753. PubMed PMID: 20030996; PubMed Central PMCID: PMCPMC2913319.

101. Ngoi CN, Price MA, Fields B, Bonventure J, Ochieng C, Mwashigadi G, et al. Dengue and chikungunya virus infections among young febrile adults evaluated for acute HIV-1 infection in coastal Kenya. PLoS One. 2016;11(12).

102. Ngoi CN, Siqueira J, Li L, Deng X, Mugo P, Graham SM, et al. The plasma virome of febrile adult Kenyans shows frequent parvovirus B19 infections and a novel arbovirus (Kadipiro virus). J Gen Virol. 2016;97(12):3359.

103. Njeru J, Henning K, Pletz MW, Heller R, Forstner C, Kariuki S, et al. Febrile patients admitted to remote hospitals in Northeastern Kenya: seroprevalence, risk factors and a clinical prediction tool for Q fever. BMC Infect Dis. 2016;16(1):244. doi: 10.1186/s12879-016-1569-0.

104. Njeru J, Tomaso H, Mertens K, Henning K, Wareth G, Heller R, et al. Serological evidence of *Francisella tularensis* in febrile patients seeking treatment at remote hospitals, northeastern Kenya, 2014–2015. New Microbes New Infect. 2017;19:62-6.

105. Ocheke O, John C, Ogbe P, Donli A, Oguche S. The febrile child: how frequent should we investigate for urinary tract infection. Niger J Paediatr. 2016;43(1):30-3.

106. Okwara FN, Obimbo EM, Wafula EM, Murila FV. Bacteraemia, urinary tract infection and malaria in hospitalised febrile children in Nairobi: is there an association? East Afr Med J. 2004;81(1):47-51. Epub 2004/04/15. doi: 10.4314/eamj.v81i1.8795. PubMed PMID: 15080516.

107. O'Meara WP, Mott JA, Laktabai J, Wamburu K, Fields B, Armstrong J, et al. Etiology of pediatric fever in western Kenya: a case–control study of falciparum malaria, respiratory viruses, and streptococcal pharyngitis. Am J Trop Med Hyg. 2015;92(5):1030-7.

108. Onoja A, Adeniji J, Olaleye O. High rate of unrecognized dengue virus infection in parts of the rainforest region of Nigeria. Acta Trop. 2016;160:39-43.

109. Onubogu UC, Anochie IC. Factors associated with bacteraemia in febrile, nonneonatal children <5 years old at the paediatric outpatient clinic of the University of Port Harcourt Teaching Hospital, Nigeria. S Afr J Child Health. 2015;9(4):124-6.

110. Onyango MD, Ghebremedhin B, Waindi EN, Kakai R, Rabsch W, Tietze E, et al. Phenotypic and genotypic analysis of clinical isolates *Salmonella* serovar Typhimurium in western Kenya. J Infect Dev Ctries. 2009;3(9):685-94. Epub 2009/10/28. doi: 10.3855/jidc.610. PubMed PMID: 19858570.

111. O'Shea MK, Clay KA, Craig DG, Matthews SW, Kao RL, Fletcher TE, et al. Diagnosis of febrile illnesses other than Ebola virus disease at an Ebola treatment unit in Sierra Leone. Clin Infect Dis. 2015;61(5):795-8.

112. Park SE, Pak GD, Aaby P, Adu-Sarkodie Y, Ali M, Aseffa A, et al. The relationship between invasive nontyphoidal *Salmonella* disease, other bacterial bloodstream infections, and malaria in Sub-Saharan Africa. Clin Infect Dis. 2016;62 Suppl 1:S23-31. Epub 2016/03/05. doi: 10.1093/cid/civ893. PubMed PMID: 26933016; PubMed Central PMCID: PMCPMC4772835.

113. Peters RP, Zijlstra EE, Schijffelen MJ, Walsh AL, Joaki G, Kumwenda JJ, et al. A prospective study of bloodstream infections as cause of fever in Malawi: clinical predictors and implications for management. Trop Med Int Health. 2004;9(8):928-34. Epub 2004/08/12. doi: 10.1111/j.1365-3156.2004.01288.x. PubMed PMID: 15304000.

114. Petit PL, Haarlem JV, Poelman M, Haverkamp MC, Wamola IA. Bacteraemia in patients presenting with fever. East Afr Med J. 1995;72(2):116-20. Epub 1995/02/01. PubMed PMID: 7796750.

115. Prabhu M, Nicholson WL, Roche AJ, Kersh GJ, Fitzpatrick KA, Oliver LD, et al. Q fever, spotted fever group, and typhus group rickettsioses among hospitalized febrile patients in northern Tanzania. Clin Infect Dis. 2011;53(4):e8-e15.

116. Preziosi M, Zimba TF, Lee K, Tomas M, Kinlin S, Nhatave-Paiva C, et al. A prospective observational study of bacteraemia in adults admitted to an urban Mozambican hospital. S Afr Med J. 2015;105(5):370-4.

117. Rabasa A, Gofama M. Urinary tract infection in febrile children in Maiduguri North Eastern Nigeria. Niger J Clin Pract. 2009;12(2).

118. Ribeiro P, Bhatt N, Ali S, Monteiro V, da Silva E, Balassiano IT, et al. Seroepidemiology of leptospirosis among febrile patients in a rapidly growing suburban slum and a flood-vulnerable rural district in Mozambique, 2012-2014: Implications for the management of fever. Int J Infect Dis. 2017;64:50-7. doi: 10.1016/j.ijid.2017.08.018.

119. Ridde V, Agier I, Bonnet E, Carabali M, Dabiré KR, Fournet F, et al. Presence of three dengue serotypes in Ouagadougou (Burkina Faso): research and public health implications. Infect Dis Poverty. 2016;5(1):23.

120. Schoepp RJ, Rossi CA, Khan SH, Goba A, Fair JN. Undiagnosed acute viral febrile illnesses, Sierra Leone. Emerg Infect Dis. 2014;20(7):1176.

121. Sigauque B, Roca A, Mandomando I, Morais L, Quinto L, Sacarlal J, et al. Community-acquired bacteremia among children admitted to a rural hospital in Mozambique. Pediatr Infect Dis J. 2009;28(2):108-13. Epub 2009/01/10. doi: 10.1097/INF.0b013e318187a87d. PubMed PMID: 19131902.

122. Socolovschi C, Mediannikov O, Sokhna C, Tall A, Diatta G, Bassene H, et al. *Rickettsia felis*–associated uneruptive fever, Senegal. Emerg Infect Dis. 2010;16(7):1140.

123. Sow SO, Diallo S, Campbell JD, Tapia MD, Keita T, Keita MM, et al. Burden of invasive disease caused by *Haemophilus influenzae* type b in Bamako, Mali: impetus for routine infant immunization with conjugate vaccine. Pediatr Infect Dis J. 2005;24(6):533-7. Epub 2005/06/04. doi: 10.1097/01.inf.0000164768.28135.0d. PubMed PMID: 15933564.

124. Ssali FN, Kamya MR, Wabwire-Mangen F, Kasasa S, Joloba M, Williams D, et al. A prospective study of community-acquired bloodstream infections among febrile adults admitted to Mulago Hospital in Kampala, Uganda. J Acquir Immune Defic Syndr Hum Retrovirol. 1998;19(5):484-9. Epub 1998/12/22. doi: 10.1097/00042560-199812150-00007. PubMed PMID: 9859962.

125. Thiga JW, Mutai BK, Eyako WK. High seroprevalence of antibodies against spotted fever and scrub typhus bacteria in patients with febrile Illness, Kenya. Emerg Infect Dis. 2015;21(4):688.

126. Thompson MG, Breiman RF, Hamel MJ, Desai M, Emukule G, Khagayi S, et al. Influenza and malaria coinfection among young children in western Kenya, 2009-2011. J Infect Dis. 2012;206(11):1674-84. Epub 2012/09/18. doi: 10.1093/infdis/jis591. PubMed PMID: 22984118; PubMed Central PMCID: PMCPMC5901689.

127. Thriemer K, Ley B, Ame SS, Deen JL, Pak GD, Chang NY, et al. Clinical and epidemiological features of typhoid fever in Pemba, Zanzibar: assessment of the performance of the WHO case definitions. PLoS One. 2012;7(12):e51823. Epub 2013/01/04. doi: 10.1371/journal.pone.0051823. PubMed PMID: 23284780; PubMed Central PMCID: PMCPMC3527440.

128. Thriemer K, Ley B, Ame S, von Seidlein L, Pak GD, Chang NY, et al. The burden of invasive bacterial infections in Pemba, Zanzibar. PLoS One. 2012;7(2):e30350. doi: 10.1371/journal.pone.0030350.

129. Waggoner J, Brichard J, Mutuku F, Ndenga B, Heath CJ, Mohamed-Hadley A, et al. Malaria and chikungunya detected using molecular diagnostics among febrile Kenyan children. Open Forum Infect Dis. 2017;4(3):ofx110. Epub 2017/07/14. doi: <https://doi.org/10.1093/ofid/ofx110>. PubMed PMID: 28702473; PubMed Central PMCID: PMCPMC5505337.

130. Walsh AL, Phiri AJ, Graham SM, Molyneux EM, Molyneux ME. Bacteremia in febrile Malawian children: clinical and microbiologic features. Pediatr Infect Dis J. 2000;19(4):312-8. Epub 2000/04/27. doi: 10.1097/00006454-200004000-00010. PubMed PMID: 10783021.

131. Woodruff P, Morrill J, Burans J, Hyams K, Woody J. A study of viral and rickettsial exposure and causes of fever in Juba, southern Sudan. Trans R Soc Trop Med Hyg. 1988;82(5):761-6.

132. Yimer E, Koopman S, Messele T, Wolday D, Newayeselassie B, Gessesse N, et al. Human leptospirosis, in Ethiopia: a pilot study in Wonji. Ethiop J Health Dev. 2004;18(1).

133. Zenebe T, Kannan S, Yilma D, Beyene G. Invasive bacterial pathogens and their antibiotic susceptibility patterns in Jimma University specialized hospital, Jimma, Southwest Ethiopia. Ethiop J Health Sci. 2011;21(1):1-8.
