# Supplementary material for: A systematic review and meta-analysis of the aetiological agents of non-malarial febrile illnesses in Africa
Source: PLoS Negl Trop Dis. 2022 Jan 24;16(1):e0010144. doi: 10.1371/journal.pntd.0010144 (PMC8812962; doi:10.1371/journal.pntd.0010144)
Supplement: S3 Table — (DOCX) [file pntd.0010144.s003.docx]

## S3 Table: Results (p values) of univariable meta-regression analyses.

| **Variable** | **Categories** | ***Salmonella* spp. (typhoidal)** | ***Salmonella* spp. (non-typhoidal)** | **Dengue** | **Chikungunya** | ***Haemophilus* spp.** | ***Staphylococcus* spp.** | ***Streptococcus* spp.** | ***Leptospira* spp.** | ***Brucella* spp.** | ***Klebsiella* spp.** |
| --- | --- | --- | --- | --- | --- | --- | --- | --- | --- | --- | --- |
| Aetiologic agents number | Single, multiple | 0.36 | 0.92 | 0.02 | 0.83 | 0.66 | <0.0001 | 0.60 | 0.04 | 0.00 | na |
| Study end date (year) | Linear | 0.91 | 0.96 | 0.02 | 0.41 | 0.11 | 0.12 | 0.15 | 0.81 | 0.01 | 0.01 |
| Study end date (year) | Categorical (decades) | 0.88 | 0.83 | 0.59 | 0.84 | 0.37 | 0.30 | 0.54 | 0.76 | 0.12 | 0.00 |
| Country | African countries | 0.74 | < 0.0001 | 0.09 | 0.76 | 1.00 | 0.99 | 0.98 | 0.02 | < 0.0001 | 0.98 |
| African region | Northern, eastern, western, central and southern Africa**^*^** | 0.76 | 0.12 | 0.83 | 0.96 | 0.94 | 0.47 | 0.87 | na | 0.41 | 0.80 |
| Study setting | Urban, rural, other (semi-, peri- and sub-urban) | 0.65 | 0.68 | 0.70 | 0.67 | 0.10 | 0.70 | 0.09 | 0.83 | 0.12 | 0.66 |
| Recruitment place | Community and healthcare facility**^†^** | na | na | 0.28 | 0.32 | na | na | na | na | na | na |
| Population status | Inpatient, inpatient and outpatient, outpatient | 0.03 | 0.60 | 0.97 | 0.60 | 0.00 | 0.32 | 0.06 | 0.02 | 0.27 | 0.81 |
| Diagnostics | Direct detection, indirect detection, direct and indirect detection | <0.0001 | na | 0.01 | 0.36 | 0.71 | 0.70 | 0.81 | 0.14 | 0.89 | 0.74 |

**^*^** Geographical regions were defined according to the United Nations Statistical Division (https://unstats.un.org/unsd/methodology/m49/).

**^†^** Healthcare facilities included primary healthcare facilities, hospitals, research units and treatment units.

na = not applicable. There was a lack of sufficient data available to estimate the coefficient in the model.
