# Supplementary material for: A systematic review and meta-analysis of the aetiological agents of non-malarial febrile illnesses in Africa
Source: PLoS Negl Trop Dis. 2022 Jan 24;16(1):e0010144. doi: 10.1371/journal.pntd.0010144 (PMC8812962; doi:10.1371/journal.pntd.0010144)
Supplement: S1 Text — (DOCX) [file pntd.0010144.s004.docx]

# S1 Text: Risk of bias assessment tool

1. Did the article address a clearly focused study topic and was there a sound study design?
2. Scientific background and rationale for study are given. YES / NO
3. Objective(s) of study is given. YES / NO
4. Setting of study is given (e.g., inpatient, outpatient, community, etc.). YES / NO
5. Location(s) of study is given (e.g., location of hospital, village, etc.). YES / NO
6. Dates or period of study recruitment/sampling are given (i.e., start and stop of data collection). YES / NO
7. Data collection methods for the data presented in the results section are described (e.g., clinical history, physical examination, sampling procedure, questionnaire administration, etc.). YES / NO
8. Was the study population selected in an acceptable way?
9. The numbers of individuals at each stage of recruitment are reported (e.g., numbers potentially eligible, examined for eligibility, confirmed eligible, included in the study, completing follow-up and analysed). YES / NO
10. Inclusion and exclusion criteria for patients are given. YES / NO
11. Sources and methods of selection of participants are given. Sample size calculation must have been included. YES / NO
12. In case of follow-up of patients in the study, period of follow up is given. YES / NO / (NA)
13. Study population characteristics are given (age, size, etc.) YES / NO
14. Reasons for non-participation at any of the stages described in 2a are given. YES / NO
15. Was a proper method used for the case definition and detection?
16. There is a defined cut-off for the fever temperature. YES / NO
17. Duration of the fever is stated. YES / NO
18. Location of body temperature measurement is stated (axillary, rectal, tympanic, oral, etc.). YES / NO
19. Accompanying clinical signs and symptoms of the infectious disease are given. YES / NO
20. The infection was diagnosed entirely by direct detection methods for pathogen(s) of interest (culture, PCR, antigen detection). YES / NO
21. The infection was diagnosed entirely by indirect detection methods for pathogen(s) of interest by rise in antibody titres. YES / NO / (NA)
22. The infection was diagnosed entirely by indirect detection methods for pathogen(s) of interest by IgM response. YES / NO / (NA)
23. Did the study recruit control populations for follow up? Control groups can be afebrile patients, but they can also be specific to the agent in question, depending on the clinical manifestations (e.g., when investigating diseases with febrile convulsions such as Lassa fever, the control group can be patients without convulsions). YES / NO

If control group is present, take a note of whether it is matched or unmatched.

1. Have robust data analyses been performed?
2. Descriptive statistics of study participants were included (e.g., demographical (age, gender, etc.), clinical (HIV status, respiratory illness, etc.) and social characteristics (occupation, wealth, income level, etc.)). YES / NO
3. Information on exposure variables is given. YES / NO / (NA)
4. Information on potential confounders is given. YES / NO
5. The authors took potential confounding factors into account, in the design of the study and/or in their analysis. Methods to deal with confounding could be excluding extraneous factors (restricted sampling), matching of confounding factors in the groups being compared or control during data analysis (e.g., by using the Mantel-Haenszel procedure for stratified data, multivariable models, etc.). YES / NO
6. The number of outcome/dependent variable events was reported (e.g., disease outcome). YES / NO
7. Unadjusted estimates of results and their precision (e.g., proportions of aetiologic agents and their 95% confidence intervals) are given. YES / NO
8. If continuous variables were categorized (e.g., age, income levels, etc.), category boundaries are reported. YES / NO / (NA)
9. Are the results well discussed?
10. Summary of key results with reference to study objectives are given. YES / NO
11. Limitations of the study are discussed (e.g., potential biases, imprecisions). YES / NO
12. Overall interpretation of results is given and compared with those from other studies. YES / NO
13. Generalizability (external validity) of the results is done. YES / NO

**NA = not applicable**
