# Supplementary material for: A systematic review and meta-analysis of the aetiological agents of non-malarial febrile illnesses in Africa
Source: PLoS Negl Trop Dis. 2022 Jan 24;16(1):e0010144. doi: 10.1371/journal.pntd.0010144 (PMC8812962; doi:10.1371/journal.pntd.0010144)
Supplement: S2 Text — (DOCX) [file pntd.0010144.s005.docx]

# S2 Text: Statistical analyses

Choropleth maps were generated using *rgdal*, *ggplot2*, and *stats* packages in R statistical environment for analysis (version 3.6.2). Boxplots of various extracted study characteristics were constructed using the *boxplot* R base function. A heat map was generated using the *heatmap* package to illustrate the prevalence of disease-specific clinical signs and symptoms. The samples tested for each agent were presented in chord diagrams created by the *chorddiag* package. The meta-regression analyses were performed using the *meta* and *metafor* packages.
