# Supplementary material for: A systematic review and meta-analysis of the aetiological agents of non-malarial febrile illnesses in Africa
Source: PLoS Negl Trop Dis. 2022 Jan 24;16(1):e0010144. doi: 10.1371/journal.pntd.0010144 (PMC8812962; doi:10.1371/journal.pntd.0010144)
Supplement: S1 Fig — (DOCX) [file pntd.0010144.s007.docx]

## S1 Fig: Distribution of the risk of bias scores for the studies included in our systematic review and subsequent meta-analysis.
