# Supplementary material for: A systematic review and meta-analysis of the aetiological agents of non-malarial febrile illnesses in Africa
Source: PLoS Negl Trop Dis. 2022 Jan 24;16(1):e0010144. doi: 10.1371/journal.pntd.0010144 (PMC8812962; doi:10.1371/journal.pntd.0010144)
Supplement: S2 Fig — The demarcating line on the x-axis (in blue) shows publication numbers before and after 2010 when the World Health Organisation recommended parasitological confirmation of suspected malaria cases (either through microscopy or rapid diagnostic tests). (DOCX) [file pntd.0010144.s008.docx]

**
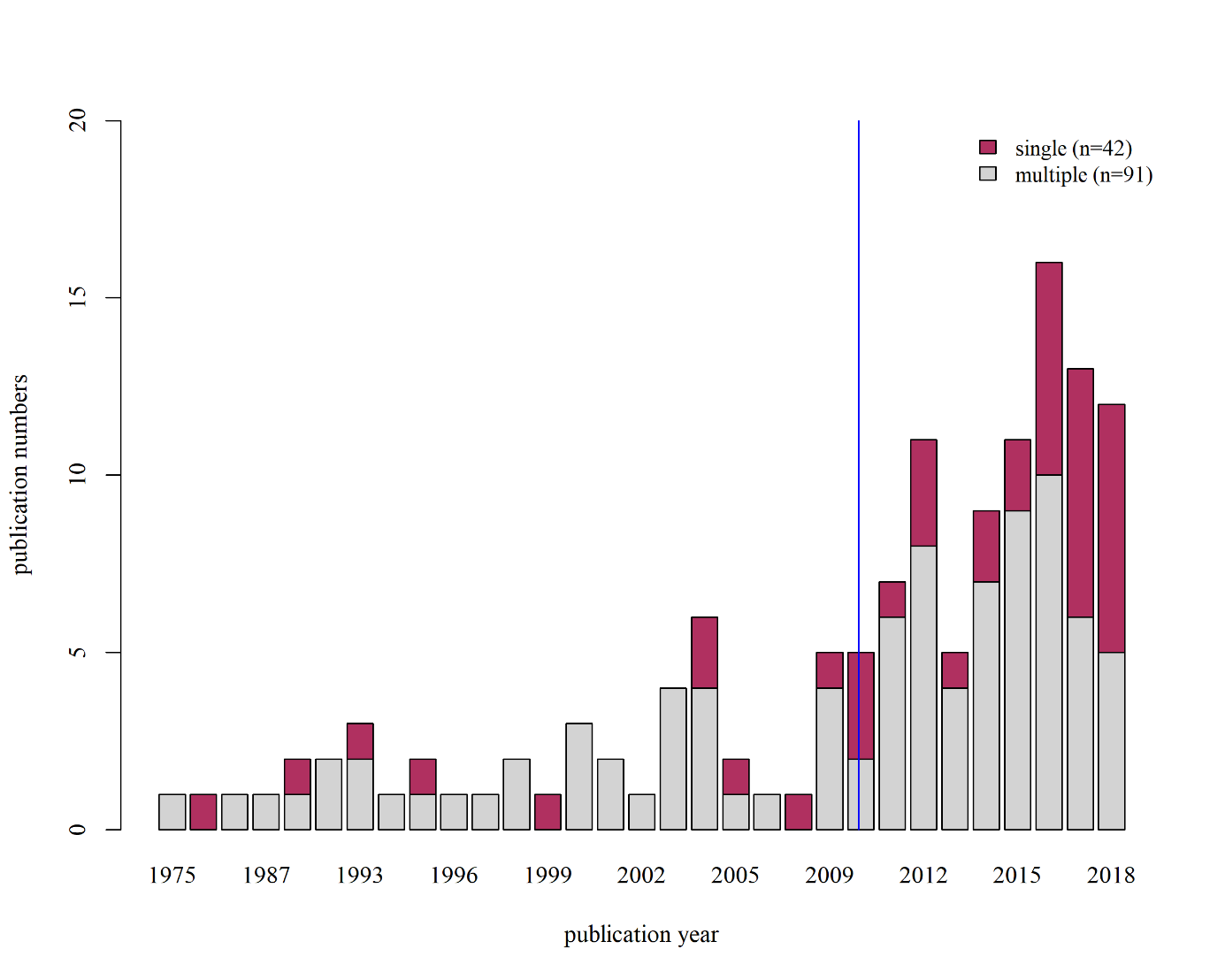
**

## S2 Fig: Distribution of included studies by publication year and type of study (whether more than one genus was investigated [multiple aetiologies study] or not [single aetiology study]). The demarcating line on the x-axis (in blue) shows publication numbers before and after 2010 when the World Health Organisation recommended parasitological confirmation of suspected malaria cases (either through microscopy or rapid diagnostic tests).
