# Supplementary figures and images for: A systematic review and meta-analysis of the aetiological agents of non-malarial febrile illnesses in Africa
Source: PLoS Negl Trop Dis. 2022 Jan 24;16(1):e0010144. doi: 10.1371/journal.pntd.0010144 (PMC8812962; doi:10.1371/journal.pntd.0010144)

**
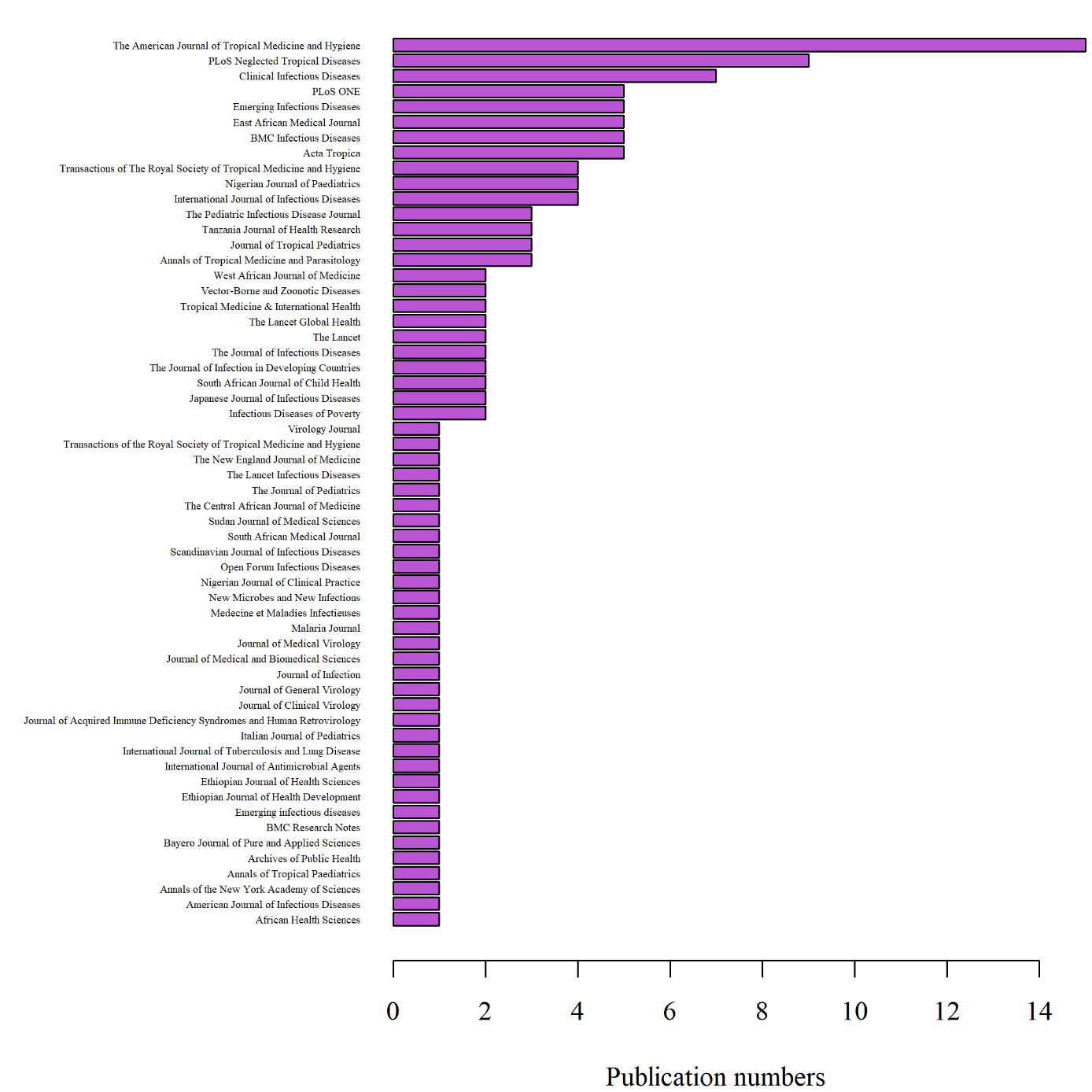
**

## S3 Fig: Journal sources of the included studies.

Supplement: S3 Fig — (DOCX) [file pntd.0010144.s009.docx]
