# Supplementary material for: A systematic review and meta-analysis of the aetiological agents of non-malarial febrile illnesses in Africa
Source: PLoS Negl Trop Dis. 2022 Jan 24;16(1):e0010144. doi: 10.1371/journal.pntd.0010144 (PMC8812962; doi:10.1371/journal.pntd.0010144)
Supplement: S4 Fig — These also represent the variables we created and used in the meta-regression analyses A. Age (in years) distributions in the study populations B. Minimum temperatures (in °C) measured in fever patients at different locations of the body C. Duration of fever (categories of 1 to 24 hrs up to 2 weeks) in the study populations (sample size) D. Status and number (sample size) of the recruited study participants E. Recruitment places of study participants (sample size) F. Study populations (sample size) recruited in different settings. (DOCX) [file pntd.0010144.s010.docx]

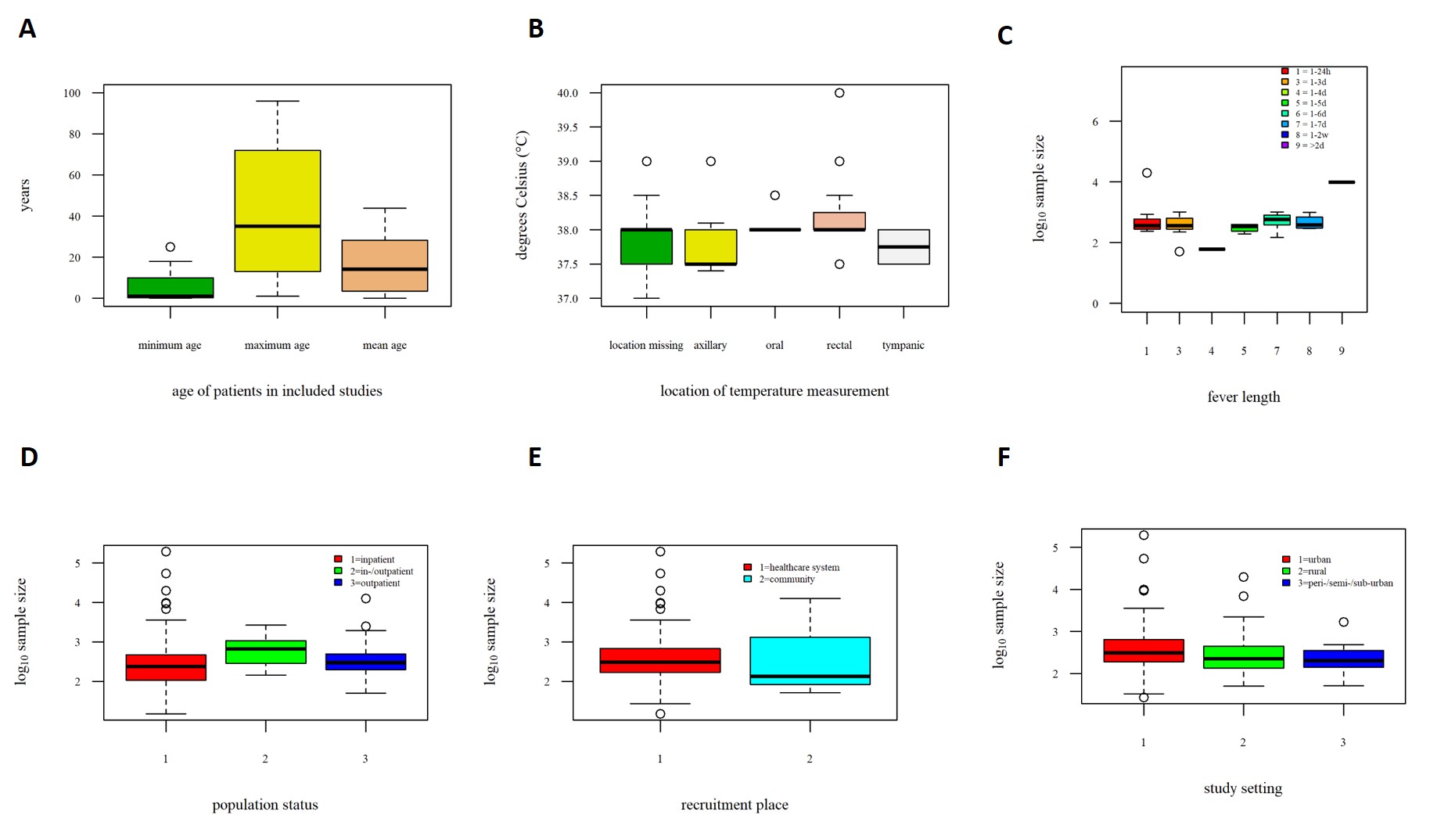
 S4 Fig: Characteristics of the included studies and the populations represented. These also represent the variables we created and used in the meta-regression analyses **A.** Age (in years) distributions in the study populations **B.** Minimum temperatures (in °C) measured in fever patients at different locations of the body **C.** Duration of fever (categories of 1 to 24 hrs up to 2 weeks) in the study populations (sample size) **D.** Status and number (sample size) of the recruited study participants **E.** Recruitment places of study participants (sample size) **F.** Study populations (sample size) recruited in different settings.
