# Supplementary material for: A systematic review and meta-analysis of the aetiological agents of non-malarial febrile illnesses in Africa
Source: PLoS Negl Trop Dis. 2022 Jan 24;16(1):e0010144. doi: 10.1371/journal.pntd.0010144 (PMC8812962; doi:10.1371/journal.pntd.0010144)
Supplement: S5 Fig — The summary estimate for typhoidal Salmonella among 293,981 patients tested was 2.0% (95% CI: 1.3–3.1). Between-study heterogeneity was significantly high (I2 = 97.8%, τ2 = 1.8). (DOCX) [file pntd.0010144.s011.docx]

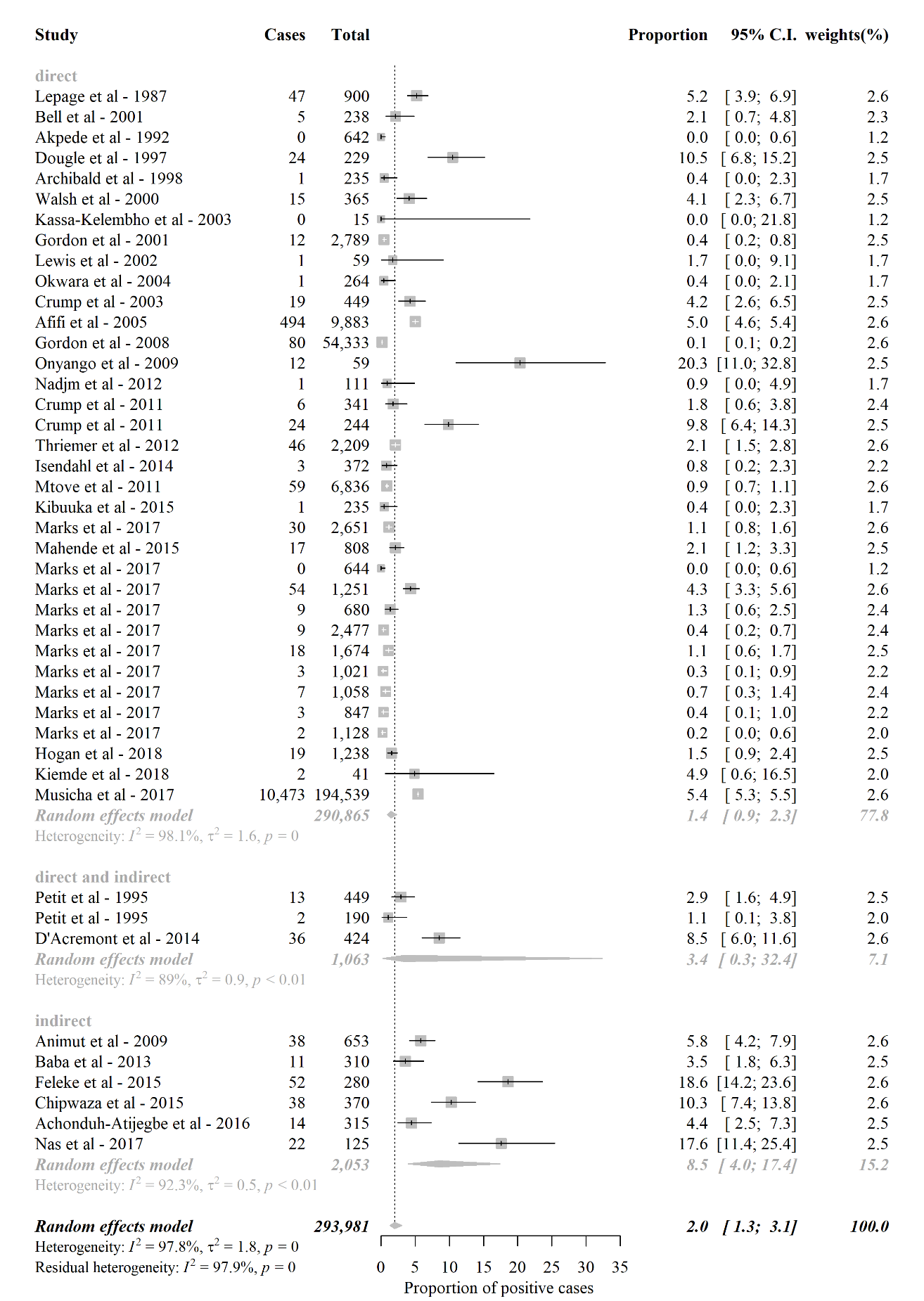


## S5 Fig: Forest plot of studies investigating typhoidal *Salmonella* (mostly comprised of *Salmonella* Typhi and *S*. Paratyphi) in order of increasing study year (referring to the end of sample collection). The summary estimate for typhoidal *Salmonella* among 293,981 patients tested was 2.0% (95% CI: 1.3-3.1). Between-study heterogeneity was significantly high (*I*^2^=97.8%, τ^2^=1.8).
