# Supplementary material for: A systematic review and meta-analysis of the aetiological agents of non-malarial febrile illnesses in Africa
Source: PLoS Negl Trop Dis. 2022 Jan 24;16(1):e0010144. doi: 10.1371/journal.pntd.0010144 (PMC8812962; doi:10.1371/journal.pntd.0010144)
Supplement: S6 Fig — The summary estimate for non-typhoidal Salmonella among 292,792 patients tested was 1.6% (95% CI. 0.8–3.3). Between-study heterogeneity was significantly high (I2 = 99.5%, τ2 = 3.5). (DOCX) [file pntd.0010144.s012.docx]

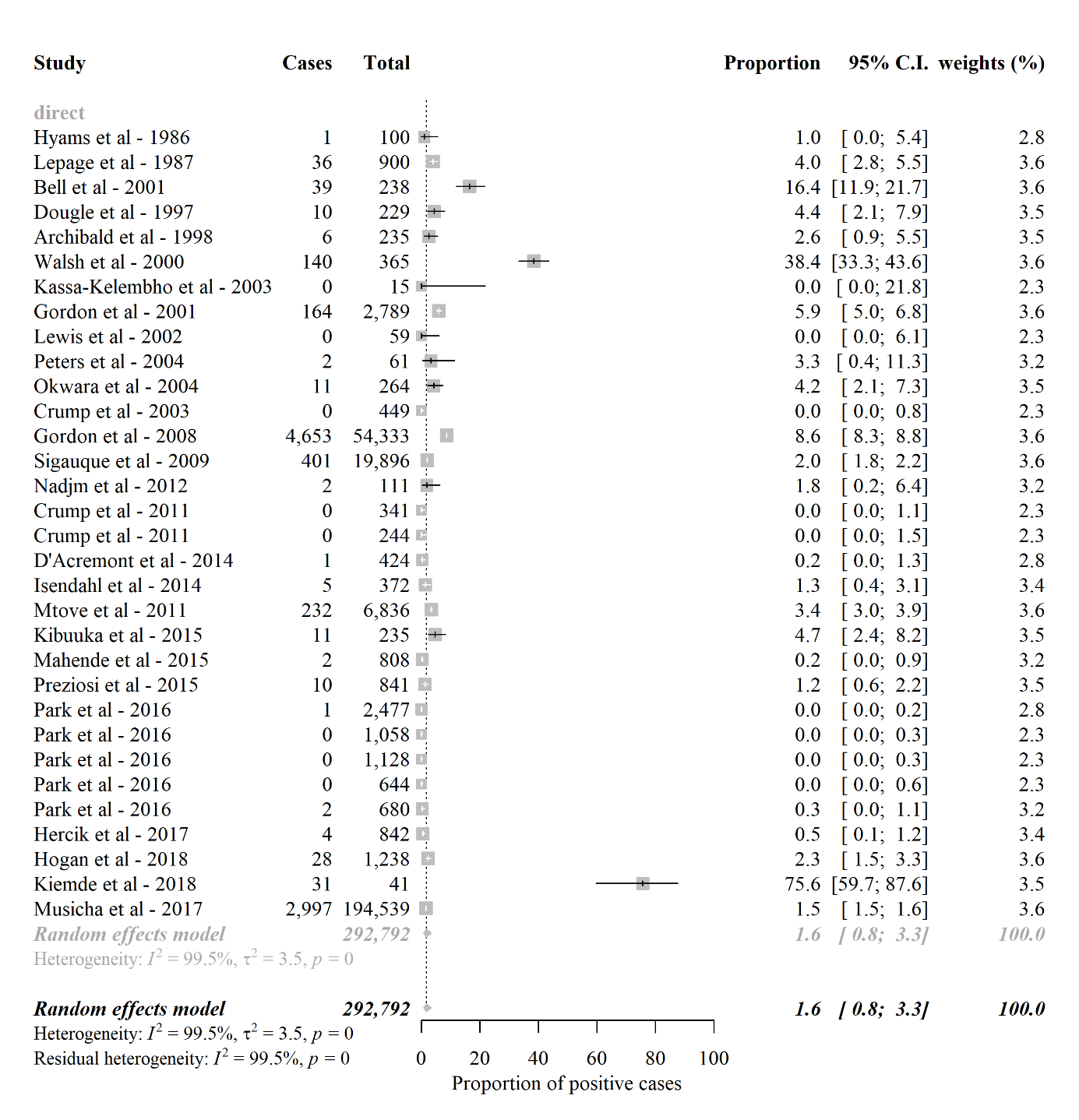


## S6 Fig: Forest plot of studies investigating non-typhoidal *Salmonella* (with identified serovars including Typhimurium, Enteritidis, Dublin, Infantis) in order of increasing study end year. The summary estimate for non-typhoidal *Salmonella* among 292,792 patients tested was 1.6% (95% CI: 0.8-3.3). Between-study heterogeneity was significantly high (*I^2^*=99.5%, τ^2^=3.5).
