# Supplementary material for: A systematic review and meta-analysis of the aetiological agents of non-malarial febrile illnesses in Africa
Source: PLoS Negl Trop Dis. 2022 Jan 24;16(1):e0010144. doi: 10.1371/journal.pntd.0010144 (PMC8812962; doi:10.1371/journal.pntd.0010144)
Supplement: S7 Fig — The summary estimate for Dengue virus among 20,112 patients tested was 8.4% (95% CI: 3.2–20.0). Between-study heterogeneity was significantly high (I2 = 98.9%, τ2 = 4.8). (DOCX) [file pntd.0010144.s013.docx]

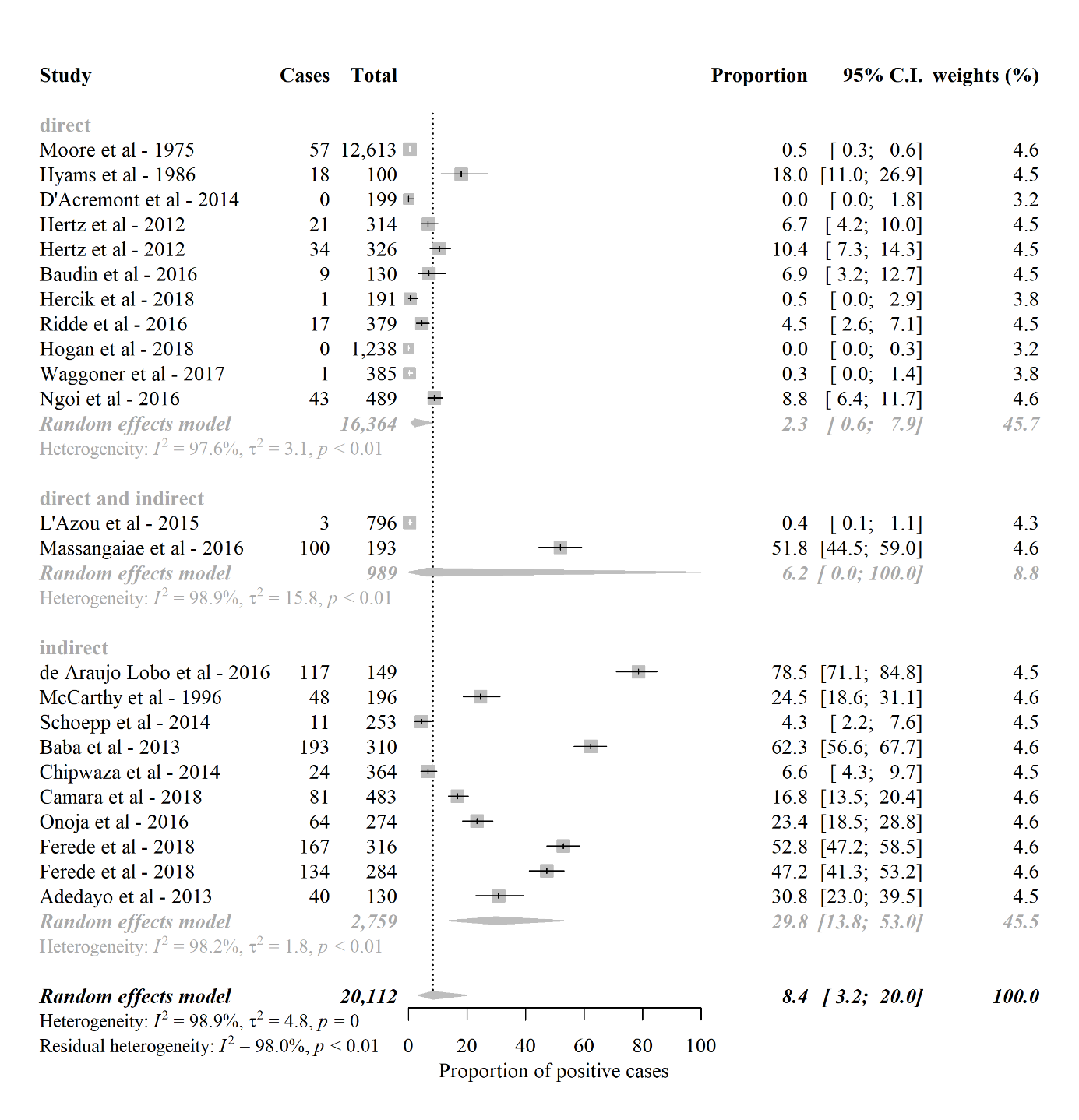


## S7 Fig: Forest plot of studies investigating Dengue virus presented by increasing study end year (Adedayo et al. lacked study end date). The summary estimate for Dengue virus among 20,112 patients tested was 8.4% (95% CI: 3.2-20.0). Between-study heterogeneity was significantly high (*I*^2^=98.9%, τ^2^=4.8).
