# Supplementary material for: A systematic review and meta-analysis of the aetiological agents of non-malarial febrile illnesses in Africa
Source: PLoS Negl Trop Dis. 2022 Jan 24;16(1):e0010144. doi: 10.1371/journal.pntd.0010144 (PMC8812962; doi:10.1371/journal.pntd.0010144)
Supplement: S8 Fig — The summary estimate for Chikungunya virus among 18,080 patients tested was 4.5% (95% CI: 1.5–12.7). Between-study heterogeneity was significantly high (I2 = 98.8%, τ2 = 3.8). (DOCX) [file pntd.0010144.s014.docx]

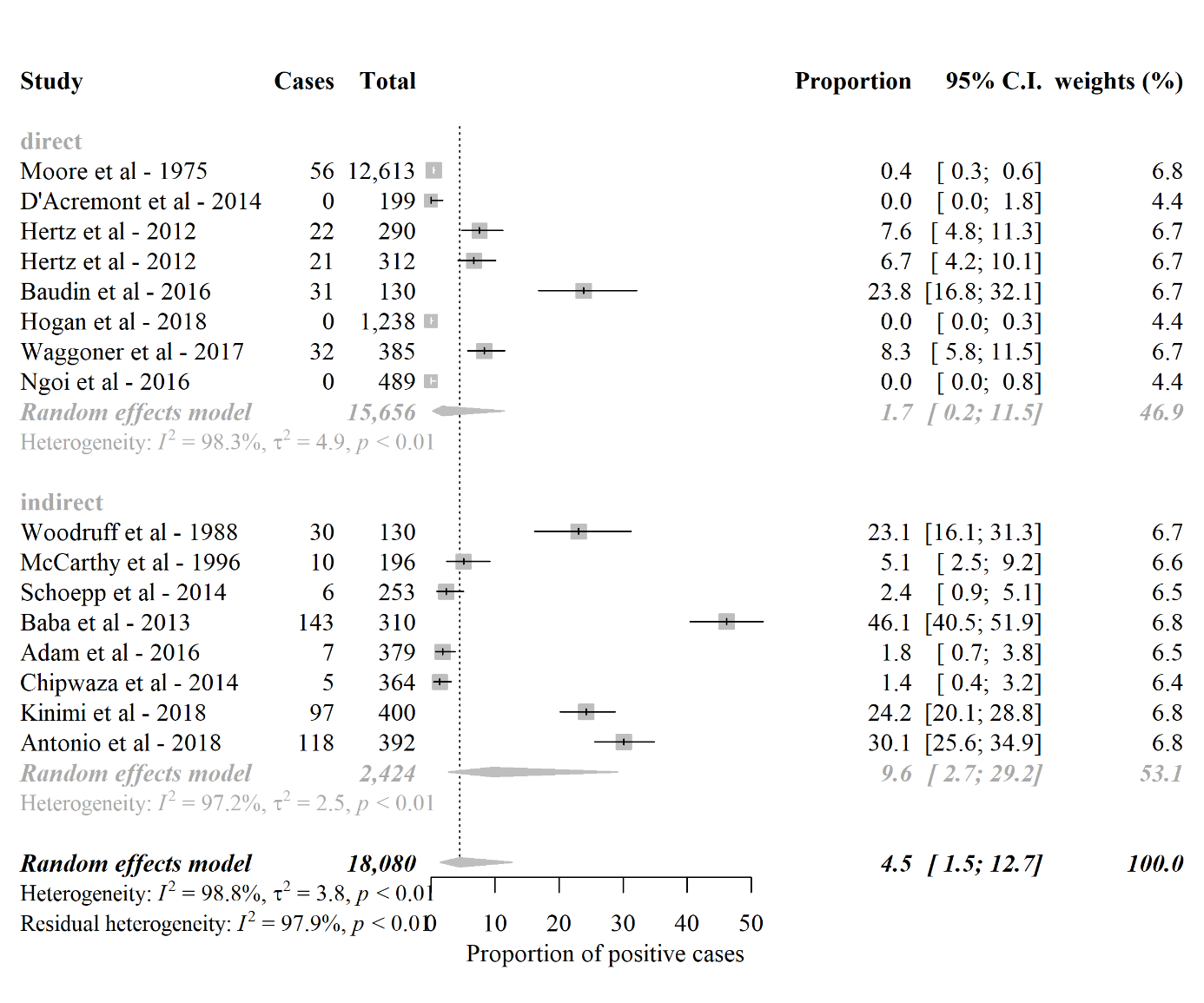


## S8 Fig: Forest plot of studies investigating Chikungunya virus presented by increasing study end year. The summary estimate for Chikungunya virus among 18,080 patients tested was 4.5% (95% CI: 1.5-12.7). Between-study heterogeneity was significantly high (*I*^2^=98.8%, τ^2^=3.8).
