# Supplementary material for: A systematic review and meta-analysis of the aetiological agents of non-malarial febrile illnesses in Africa
Source: PLoS Negl Trop Dis. 2022 Jan 24;16(1):e0010144. doi: 10.1371/journal.pntd.0010144 (PMC8812962; doi:10.1371/journal.pntd.0010144)
Supplement: S9 Fig — The summary estimate for Haemophilus spp. among 240,446 patients tested was 1.4% (95% CI: 0.5–3.6). Between-study heterogeneity was significantly high (I2 = 99.5%, τ2 = 5.3). (DOCX) [file pntd.0010144.s015.docx]

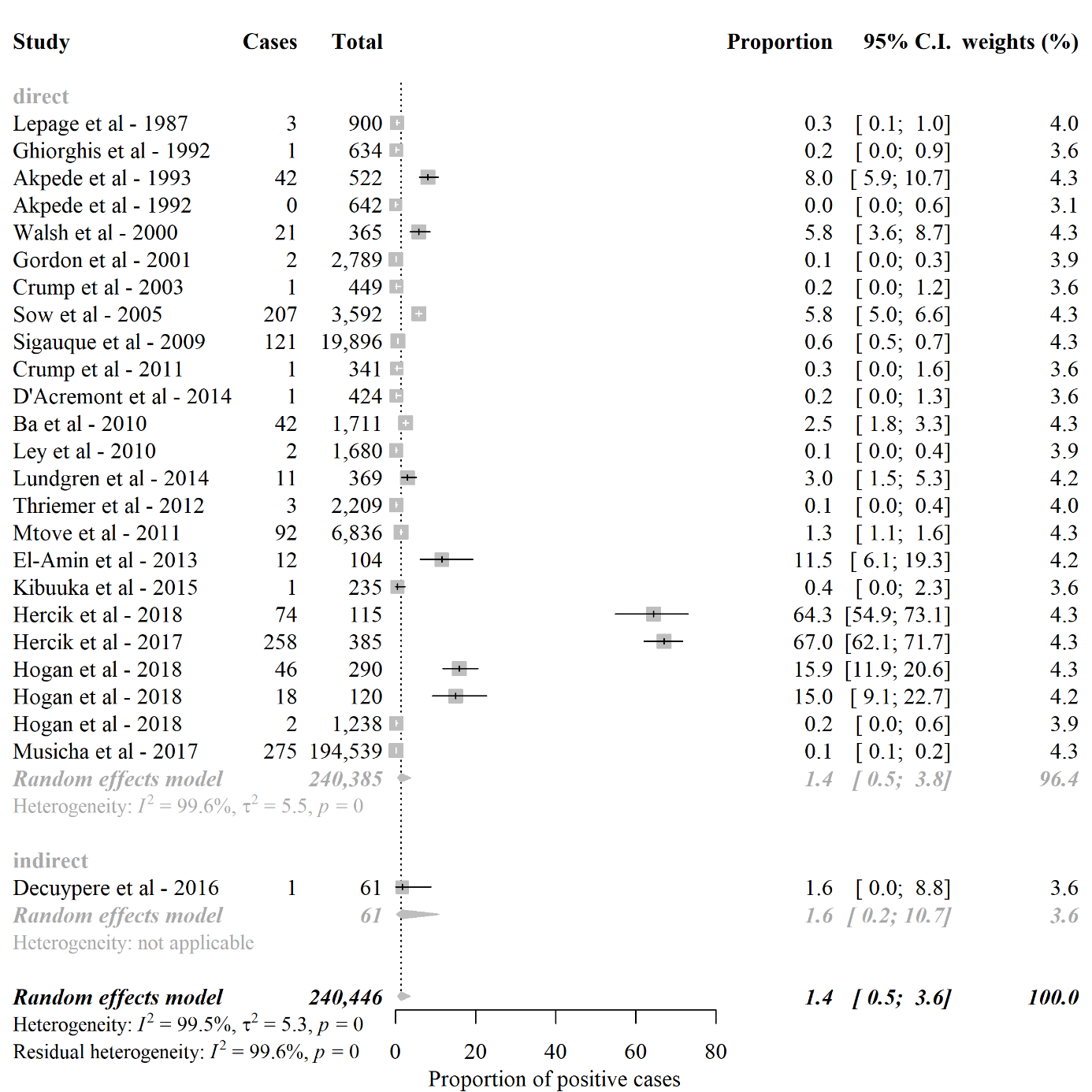


## S9 Fig: Forest plot of studies investigating *Haemophilus* spp. (with identified species including *H. influenzae* and *H. parainfluenzae*) presented by increasing study end year. The summary estimate for *Haemophilus* spp. among 240,446 patients tested was 1.4% (95% CI: 0.5-3.6). Between-study heterogeneity was significantly high (*I*^2^=99.5%, τ^2^=5.3).
