# Supplementary material for: A systematic review and meta-analysis of the aetiological agents of non-malarial febrile illnesses in Africa
Source: PLoS Negl Trop Dis. 2022 Jan 24;16(1):e0010144. doi: 10.1371/journal.pntd.0010144 (PMC8812962; doi:10.1371/journal.pntd.0010144)
Supplement: S10 Fig — The summary estimate for Staphylococcus spp. among 250,500 patients tested was 2.1% (95% CI: 1.4–3.3). Between-study heterogeneity was significantly high (I2 = 98.7%, τ2 = 2.1). (DOCX) [file pntd.0010144.s016.docx]

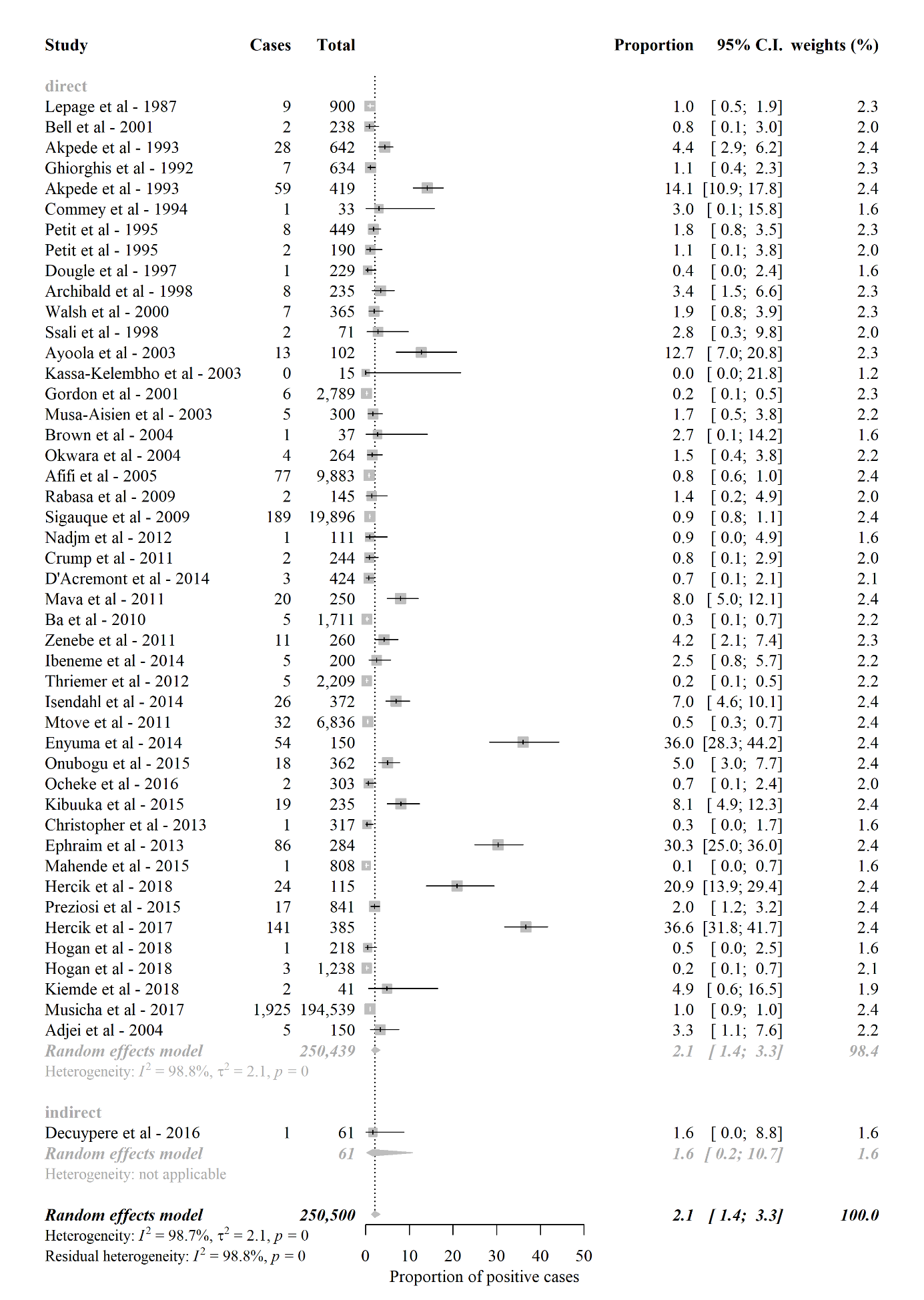


## S10 Fig: Forest plot of studies investigating *Staphylococcus* spp. (with identified species including *S. aureus* and *S. epidermidis)* presented by increasing study end year (Adjei et al. lacked study end date). The summary estimate for *Staphylococcus* spp. among 250,500 patients tested was 2.1% (95% CI: 1.4-3.3). Between-study heterogeneity was significantly high (*I*^2^=98.7%, τ^2^=2.1).
