# Supplementary material for: A systematic review and meta-analysis of the aetiological agents of non-malarial febrile illnesses in Africa
Source: PLoS Negl Trop Dis. 2022 Jan 24;16(1):e0010144. doi: 10.1371/journal.pntd.0010144 (PMC8812962; doi:10.1371/journal.pntd.0010144)
Supplement: S11 Fig — Between-study heterogeneity was significantly high (I2 = 99.1%, τ2 = 2.9). (DOCX) [file pntd.0010144.s017.docx]

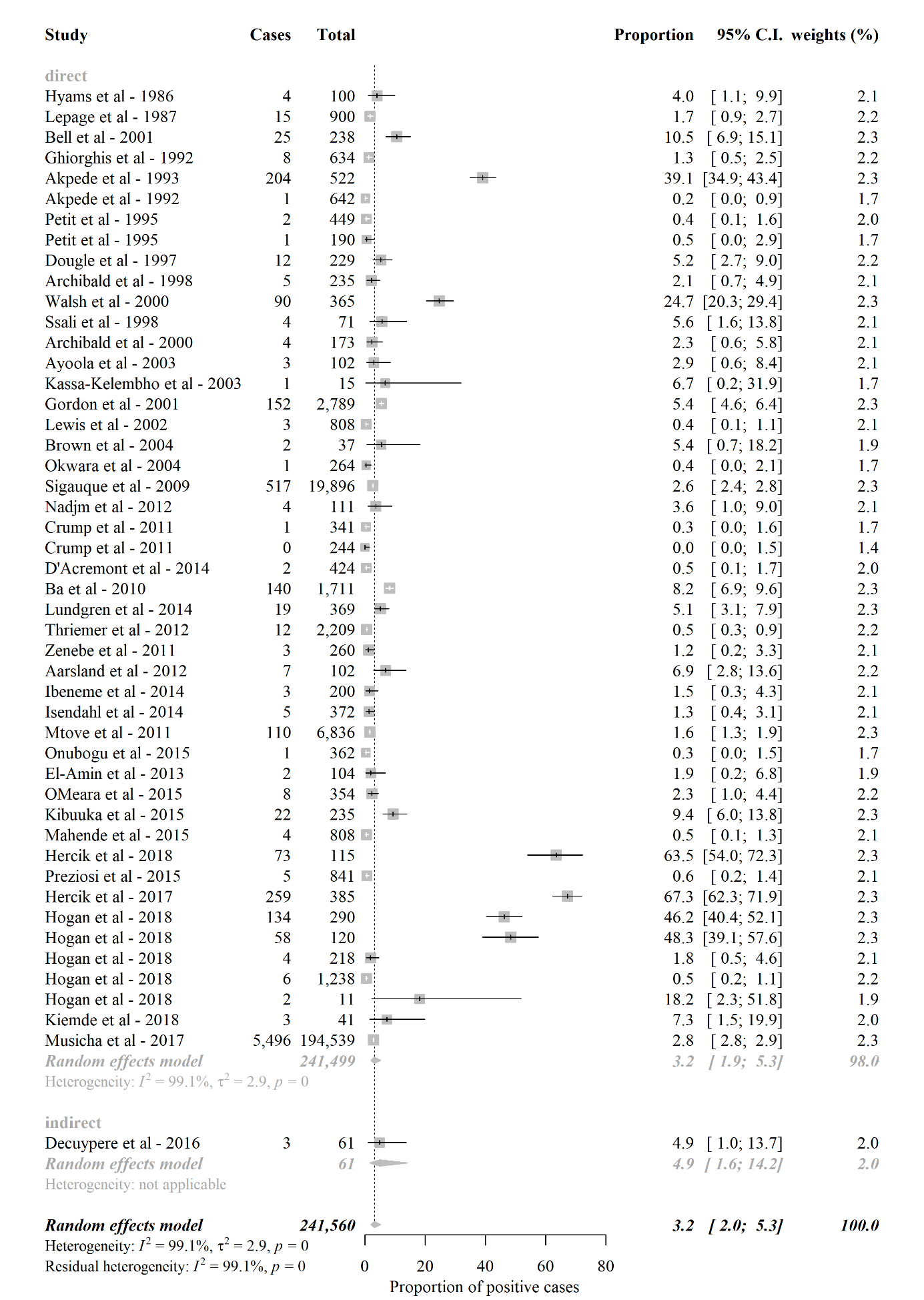


## S11 Fig: Forest plot of studies investigating *Streptococcus* spp. presented by increasing study end year. The summary estimate for *Streptococcus* spp. among 241,560 patients tested was 3.2% (95% CI: 2.0-5.3). Between-study heterogeneity was significantly high (*I*^2^=99.1%, τ^2^=2.9).
