# Supplementary material for: A systematic review and meta-analysis of the aetiological agents of non-malarial febrile illnesses in Africa
Source: PLoS Negl Trop Dis. 2022 Jan 24;16(1):e0010144. doi: 10.1371/journal.pntd.0010144 (PMC8812962; doi:10.1371/journal.pntd.0010144)
Supplement: S12 Fig — The summary estimate for Leptospira spp. among 7,182 patients tested was 3.2% (95% CI: 1.1–8.9). Between-study heterogeneity was significantly high (I2 = 95.5%, τ2 = 4.2). (DOCX) [file pntd.0010144.s018.docx]

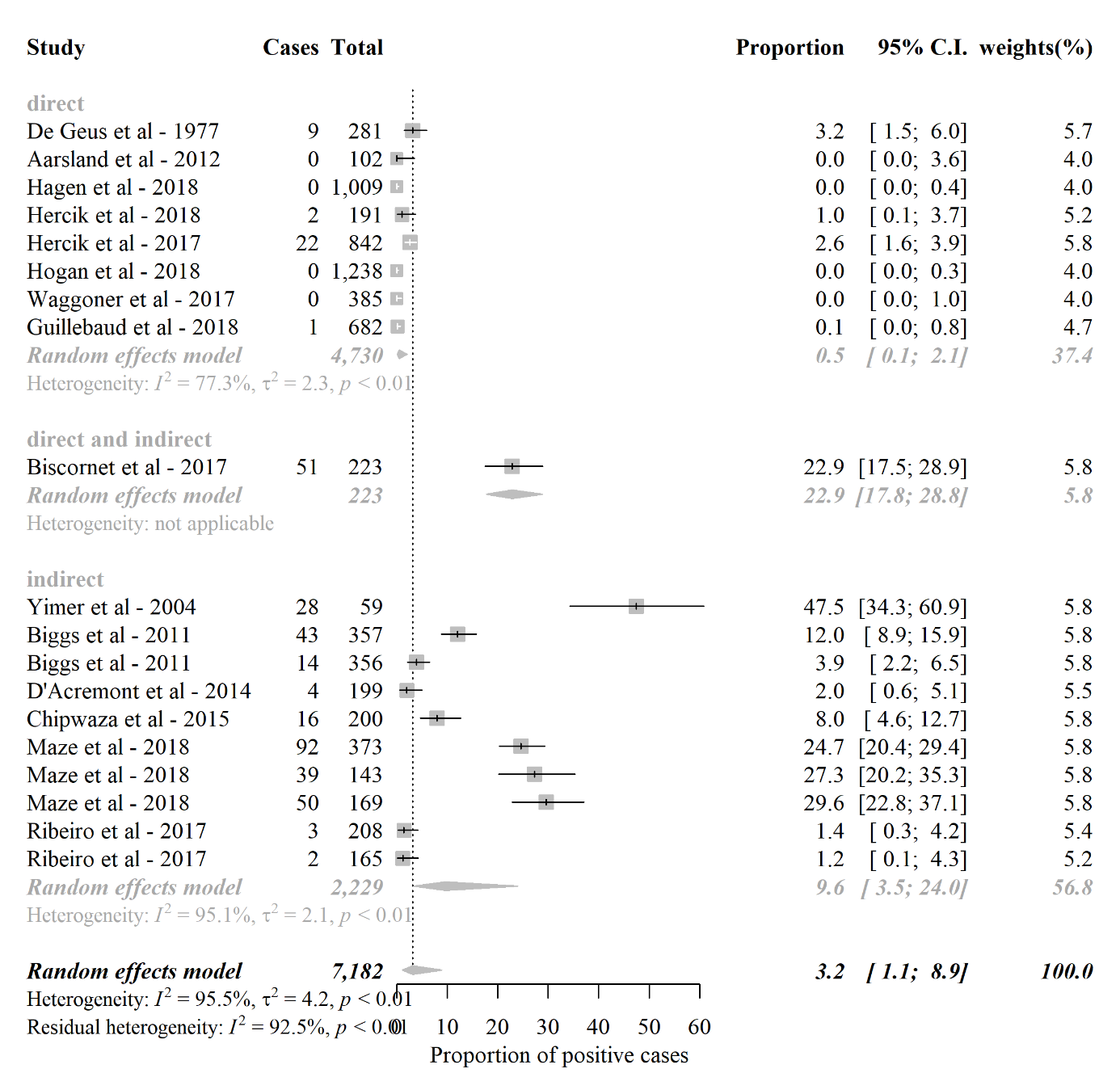


## S12 Fig: Forest plot of studies investigating *Leptospira* spp. presented by increasing study end year. The summary estimate for *Leptospira* spp. among 7,182 patients tested was 3.2% (95% CI: 1.1-8.9). Between-study heterogeneity was significantly high (*I*^2^=95.5%, τ^2^=4.2).
