# Supplementary material for: A systematic review and meta-analysis of the aetiological agents of non-malarial febrile illnesses in Africa
Source: PLoS Negl Trop Dis. 2022 Jan 24;16(1):e0010144. doi: 10.1371/journal.pntd.0010144 (PMC8812962; doi:10.1371/journal.pntd.0010144)
Supplement: S13 Fig — The summary estimate for Brucella spp. among 16,717 patients tested was 3.5% (95% CI: 1.7–7.1). Between-study heterogeneity was significantly high (I2 = 94.6%, τ2 = 1.4). (DOCX) [file pntd.0010144.s019.docx]

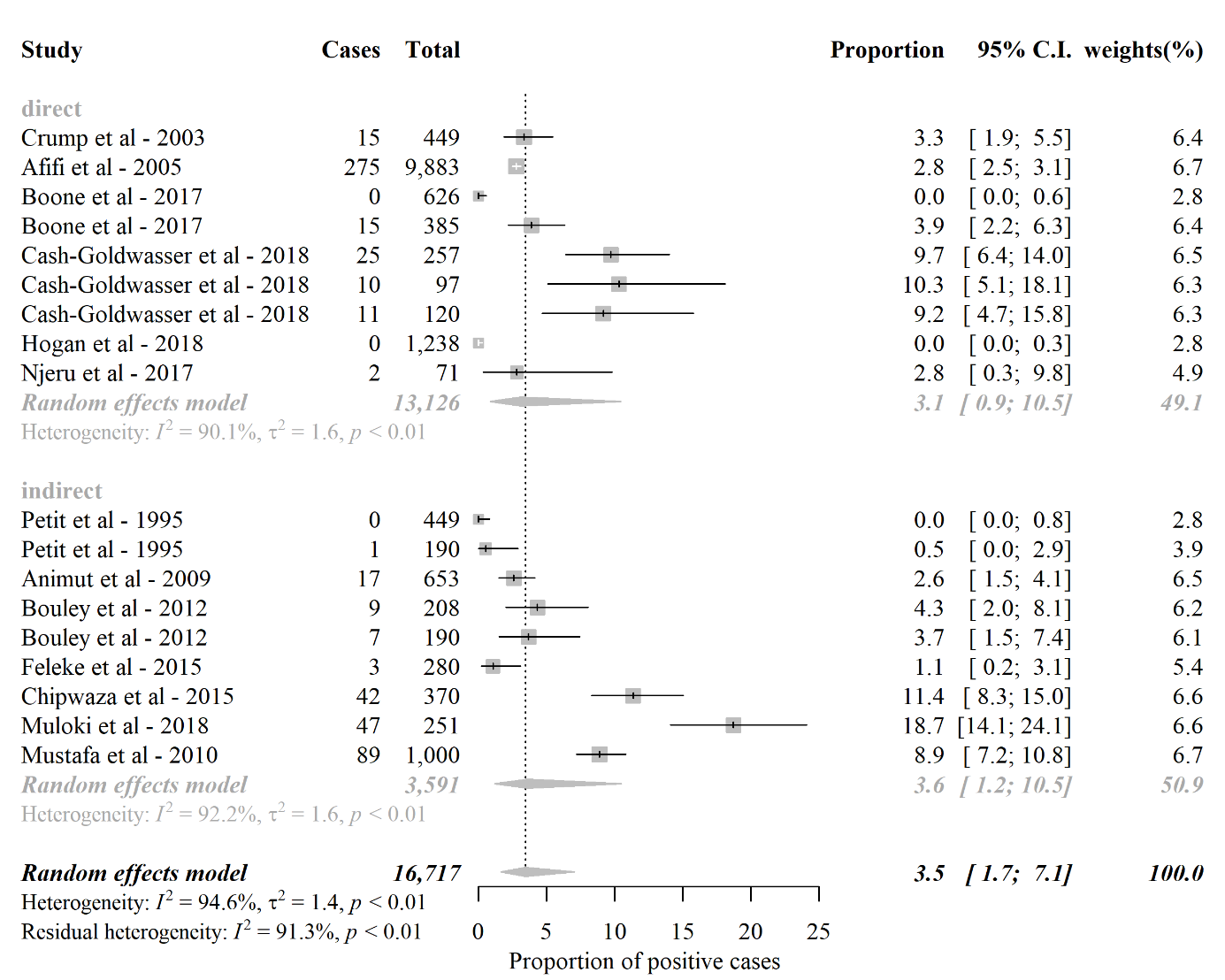


## S13 Fig: Forest plot of studies investigating *Brucella* spp. presented by increasing study end year (Mustafa et al. lacked study end date). The summary estimate for *Brucella* spp. among 16,717 patients tested was 3.5% (95% CI: 1.7-7.1). Between-study heterogeneity was significantly high (*I*^2^=94.6%, τ^2^=1.4).
