# Supplementary material for: A systematic review and meta-analysis of the aetiological agents of non-malarial febrile illnesses in Africa
Source: PLoS Negl Trop Dis. 2022 Jan 24;16(1):e0010144. doi: 10.1371/journal.pntd.0010144 (PMC8812962; doi:10.1371/journal.pntd.0010144)
Supplement: S14 Fig — The summary estimate for Klebsiella spp. among 226,762 patients tested was 1.8% (95% CI: 1.0–3.1). Between-study heterogeneity was significantly high (I2 = 98.9%, τ2 = 2.3). (DOCX) [file pntd.0010144.s020.docx]

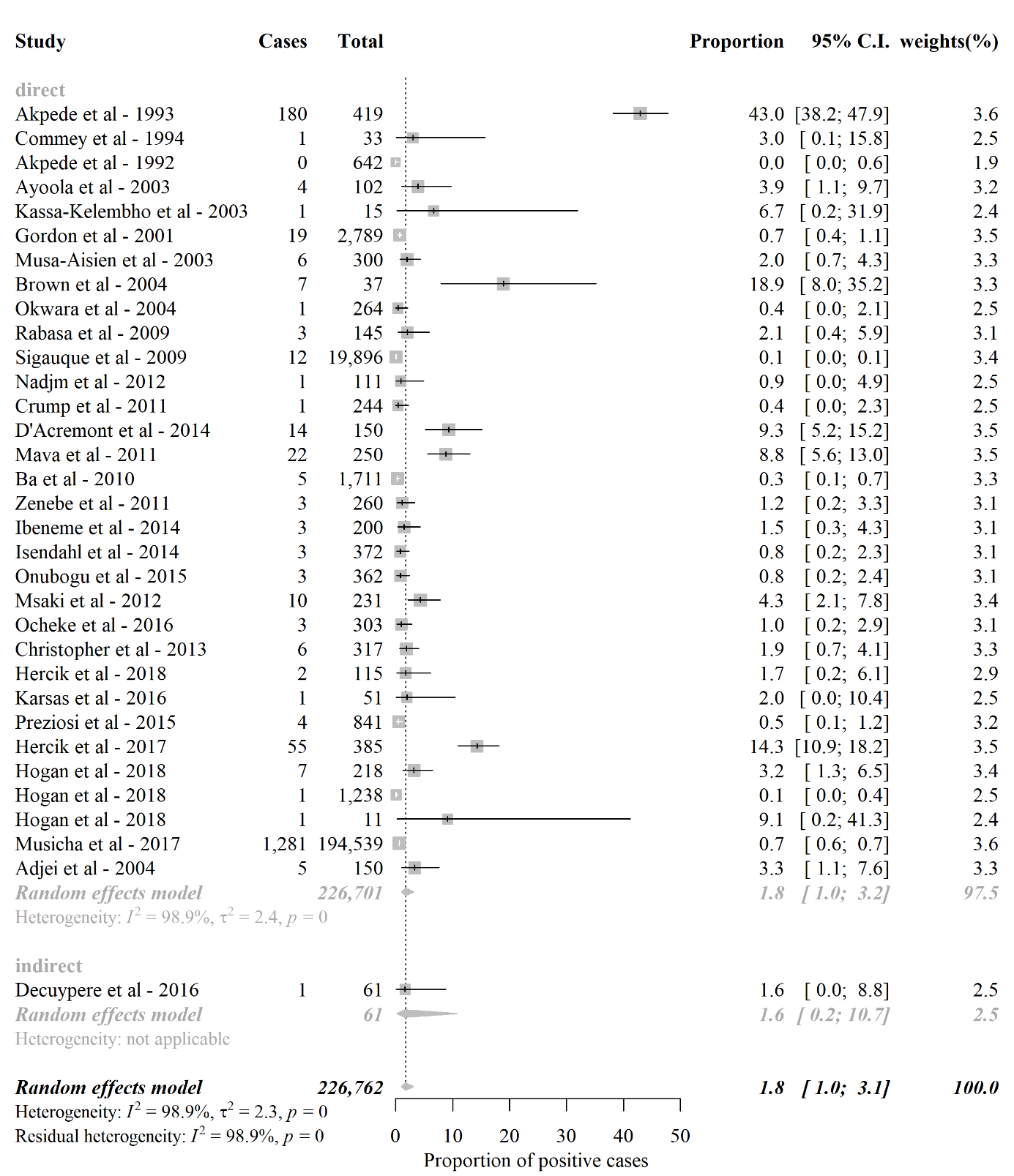


## S14 Fig: Forest plot of studies investigating *Klebsiella* spp. presented by increasing study end year (Adjei et al. lacked study end date). The summary estimate for *Klebsiella* spp. among 226,762 patients tested was 1.8% (95% CI: 1.0-3.1). Between-study heterogeneity was significantly high (*I*^2^=98.9%, τ^2^=2.3).
